# Comparative analysis of the transcriptomes of EDL, psoas, and soleus muscles from mice

**DOI:** 10.1186/s12864-020-07225-2

**Published:** 2020-11-19

**Authors:** Pabodha Hettige, Uzma Tahir, Kiisa C. Nishikawa, Matthew J. Gage

**Affiliations:** 1 Department of Chemistry, University of Massachusetts Lowell, Lowell, MA 01854 USA; 2 UMass Movement Center, University of Massachusetts Lowell, Lowell, MA 01854 USA; 3 Department of Biological Sciences, Northern Arizona University, Flagstaff, AZ 86011 USA

**Keywords:** RNA sequencing, Fast-twitch muscle, Slow-twitch muscle, Transcriptome, Muscle specialization, Differential gene expression

## Abstract

**Background:**

Individual skeletal muscles have evolved to perform specific tasks based on their molecular composition. In general, muscle fibers are characterized as either fast-twitch or slow-twitch based on their myosin heavy chain isoform profiles. This approach made sense in the early days of muscle studies when SDS-PAGE was the primary tool for mapping fiber type. However, Next Generation Sequencing tools permit analysis of the entire muscle transcriptome in a single sample, which allows for more precise characterization of differences among fiber types, including distinguishing between different isoforms of specific proteins. We demonstrate the power of this approach by comparing the differential gene expression patterns of extensor digitorum longus (EDL), psoas, and soleus from mice using high throughput RNA sequencing.

**Results:**

EDL and psoas are typically classified as fast-twitch muscles based on their myosin expression pattern, while soleus is considered a slow-twitch muscle. The majority of the transcriptomic variability aligns with the fast-twitch and slow-twitch characterization. However, psoas and EDL exhibit unique expression patterns associated with the genes coding for extracellular matrix, myofibril, transcription, translation, striated muscle adaptation, mitochondrion distribution, and metabolism. Furthermore, significant expression differences between psoas and EDL were observed in genes coding for myosin light chain, troponin, tropomyosin isoforms, and several genes encoding the constituents of the Z-disk.

**Conclusions:**

The observations highlight the intricate molecular nature of skeletal muscles and demonstrate the importance of utilizing transcriptomic information as a tool for skeletal muscle characterization.

**Supplementary Information:**

The online version contains supplementary material available at 10.1186/s12864-020-07225-2.

## Background

Muscle sarcomere is a complex network of proteins that work together to generate force. Specific fiber types have evolved to express a unique array of proteins according to the tasks that muscles perform [1–3]. The basic principles associated with muscle contraction were established with the development of the sliding filament model [4, 5]. However, there remain many unanswered questions associated with muscle function and the differences among skeletal muscle fiber types [2, 6, 7], even after close to a century of work. Transcriptomic variability among different types of skeletal muscle is one particular area that has been poorly characterized. A complete understanding of these differences will not only help improve fiber type characterization but will also provide the necessary tools to characterize transcriptome-level changes associated with age or muscle-based diseases.

Muscle fibers are classified at the physiological level as either fast-twitch or slow-twitch, based on their contractile properties. Myosin heavy chain isoform composition is the most common technique used in muscle fiber typing and is associated with contractile efficiency and energy metabolism [8]. Metabolic properties of muscle fibers associated with the mitochondrial structure and mitochondrial-associated enzyme content [6] determine the degree of aerobic (oxidative) and anaerobic (glycolytic) capacity [9], resulting in four primary categories of muscle; slow-twitch oxidative type 1, fast-twitch oxidative glycolytic type 2A, fast-twitch glycolytic type 2X, and fast-twitch glycolytic type 2B [6, 8, 10–12]. Several other structural and physiological characteristics, including mitochondrial composition [2, 13, 14], energetic substrate availability [2, 14], Z-line thickness [2, 14], and myoplasmic Ca^2+^ concentration and Ca^2+^ affinity of regulatory proteins [15] follow the same primary fiber type classification. While this approach provides a high-level characterization of muscle fiber type, it does not provide insights into the more subtle but important differences in gene expression that occur in different types of skeletal muscles. The majority of skeletal muscles contain a combination of fast- and slow-twitch fibers [8], resulting in characteristics of both fiber types, although they are generally classified based on the predominant myosin heavy chain isoform as either fast-twitch or slow-twitch.

Skeletal muscles vary enormously in embryonic origins, shapes, and functional roles [3]. The diversity of structural and functional constituents of myofibers makes skeletal muscles highly adaptable to functional demands [2, 16, 17]. This diversity has been observed in metabolic profiles [2, 14] and isotonic shortening velocities [2, 18, 19], despite fundamental slow- or fast-twitch characteristics remaining well preserved. However, these studies have not been correlated with differential gene expression patterns to develop a complete molecular understanding of how gene expression drives the physiology. This is an important gap in knowledge that is critical to understanding the etiology of sarcopenia, dynapenia, myopathy, muscle injuries, and other muscle diseases.

Muscle diseases are generally linked to a heritable genetic mutation or metabolic dysfunction and interestingly are often localized to a specific muscle(s) rather than targeting all muscles with a similar fiber type or architecture [16, 20]. For example, Duchenne muscular dystrophy (DMD) predominantly affects fast muscle fibers in the body but does not affect the head muscles [20]. Because susceptibility of a particular muscle to a disease phenotype is likely a function of gene expression, lack of data on gene expression differences between muscle types prevents a deeper understanding of musculoskeletal diseases. One of the most common approaches for assessing transcriptomic profiles is RNA Sequencing (RNA-Seq), which quantitatively determines the expression level of all transcripts within a particular tissue. This approach has been largely overlooked as a tool to explore transcriptomic variability in muscles. One of the pioneering studies was recently published by Terry et al. [3], who conducted a comprehensive study of the transcriptomic diversity of skeletal, smooth, and cardiac muscle tissues in mice and rats. This study demonstrated that there are two main clusters of genes in skeletal muscles that exhibited transcriptomic variability that correlated with fast-twitch and slow-twitch muscle phenotypes. However, one of the key findings of this study was that muscle-specific transcriptomic variability is more complex than an orthogonal classification of fiber types, and it is too simplistic to just refer to muscles by their prominent phenotype. Skeletal muscle classification should extend beyond the fiber type associated behavior, and take into account the physiological, metabolic, morphological, and developmental diversity of different muscles.

Comparative literature focusing on biophysical characteristics of skeletal muscles generally use representative muscles composed of primarily of either fast-twitch or slow-twitch fibers [21–23]. Much of the work using isolated muscles and muscle fibers has focused on EDL and soleus fibers as they represent fast-twitch and slow-twitch muscles [6, 24, 25]. Psoas muscle is generally classified as a fast-twitch muscle based on physiological studies, but the exact fiber composition of this muscle is less clear since the myosin heavy chain (Myh) distribution pattern differs among studies [26, 27]. Since EDL and psoas have been categorized as fast muscles, we predicted that the transcriptome of mouse psoas muscle would more closely resemble the transcriptome of the EDL muscle, while soleus would have a distinctly different gene expression pattern. In this study, we test this hypothesis using RNA-Seq and demonstrate that many of the gene clusters analyzed show similar patterns of up- and down-regulation in the psoas and EDL transcriptomes and these patterns differ from soleus. However, there are specific gene clusters where either psoas or EDL muscles more closely resemble the expression pattern of soleus muscles. Our results highlight the importance of transcriptome-level comparisons to gain insights into the molecular characteristics of skeletal muscles, that were not possible to obtain using physiological studies.

## Results

**Fig. 1:**
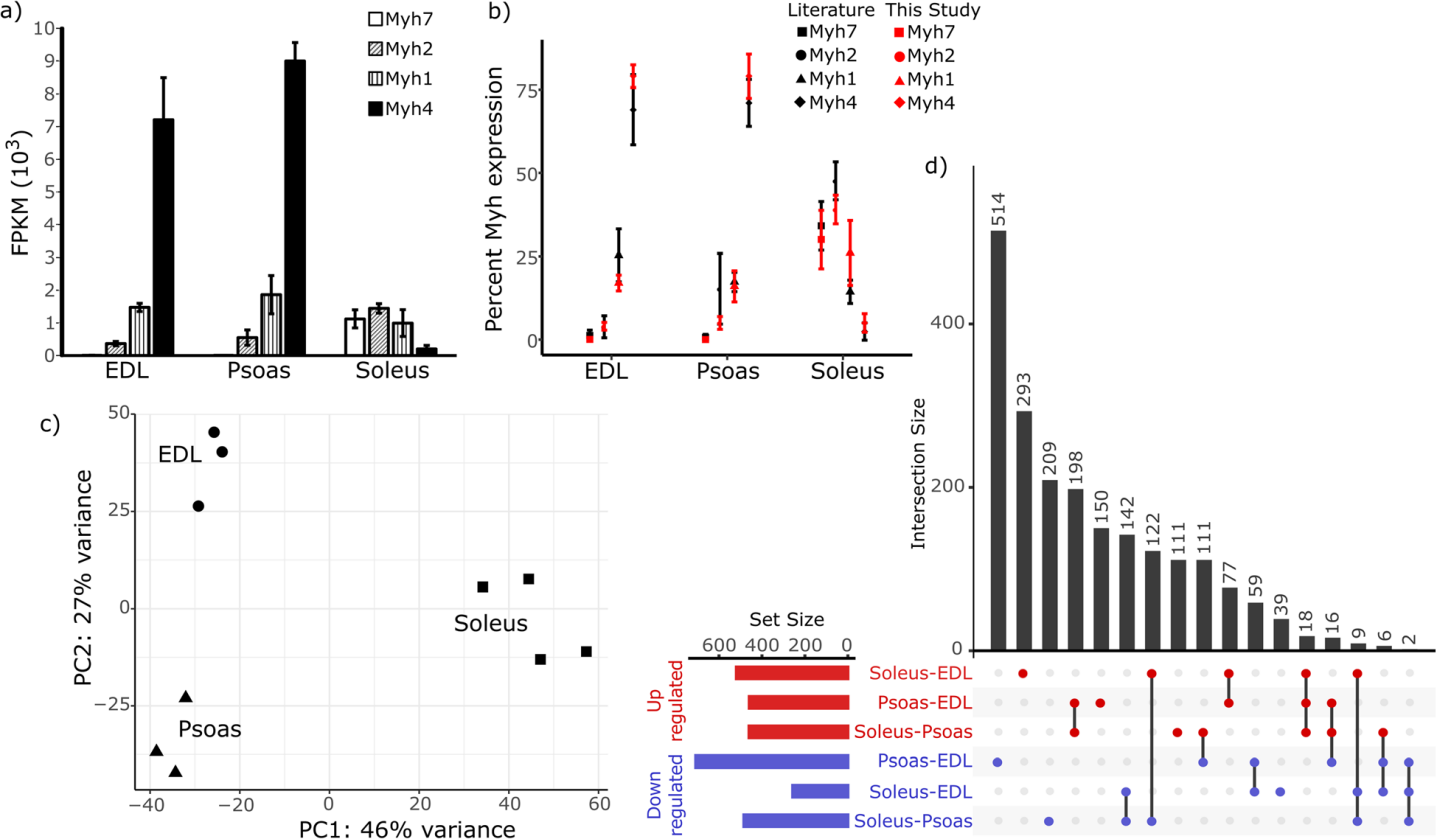
EDL, psoas, and soleus constitute unique expression signatures. **a** Expression of the four primary myosin isoforms in EDL, psoas, and soleus. Both EDL and psoas predominantly express Myh-2B (Myh4) isoform, while soleus expresses nearly equivalent levels of Myh-1(Myh7), Myh-2A(Myh2), and Myh-2X(Myh1). **b** Percent Myh isoform expression in mouse EDL, psoas, and soleus from the present study (red) agree well with the proteomics and transcriptomics data gathered from the literature (black). Percent Myh isoform expression was calculated as a fraction of total FPKM values of Myh1, Myh2, Myh4, and Myh7, and compared to the data reported by previous publications using t-tests. Data used to carry out the statistical analysis, and the results are shown in Table S2 in SI. At the 95% significant level, no significant expression difference was observed between the two datasets (**c**) PCA after filtering-out marginally expressed genes (see methods for the selection criteria used). PC1 identifies 46% of the expression variability among genes and creates two major clusters separating fast muscles (EDL and psoas) from slow muscle (soleus). PC2 accounts for 27% of the variance and separates EDL, psoas, and soleus into three distinct clusters. **d** UpSet plot showing the number of unique and overlapping genes between the up and down-regulated genes identified by the pairwise comparison of EDL, psoas, and soleus. The total number of uniquely differentially expressed genes between two fast muscles is greater than that of the fast to slow comparisons

Gene expression profiles of EDL, psoas, and soleus collected from RNA-seq were studied to understand the transcriptomic variation among muscles. The myosin heavy chain (Myh) composition of these muscles has been characterized in several different studies [3, 8, 13, 24, 26–29], providing a robust data set for validating our RNA-Seq analysis. There are four predominant myosin isoforms that are typically used for isoform typing of muscle fibers, namely Myh-2X, Myh-2A, Myh-2B, and Myh-1, which are encoded by the Myh1, 2, 4, and 7 genes respectively. The expression percentages of these four genes in EDL, psoas, and soleus were determined as a fraction of total normalized reads counts in fragments per kilobase million (FPKM) and compared to published percent expression levels (Fig. 1b). The calculated percentages of myosin heavy chain isoforms in each muscle showed no significant difference compared to the transcriptomic and proteomic data gathered from the literature when compared using a 95% significant level (Table S2 in SI). The percent expression levels of the major myosin heavy chain isoforms in EDL and psoas are similar (~ 79% Myh-2B; ~ 16% Myh-2X and ~ 4% Myh-2A) with Myh4 exhibiting the highest transcript level of any isoform in both EDL and psoas, consistent with their classification as fast-twitch muscles. In contrast, soleus expresses a mixture of both fast and slow myosin isoforms (5% Myh-2B, 26% Myh-2X, 39% Myh-2A, and 30% Myh-1), which is similar to the percentages observed in previous transcriptomics and proteomics experiments (Fig. 1a-b, Table S2 in SI).

### EDL and psoas exhibit different transcriptomic patterns

Pairwise comparison of gene expression profiles in EDL, psoas, and soleus was carried out using DESeq2 and differentially expressed genes among the muscles were identified. At a 99% significance level, the comparison of psoas vs. EDL identified 1227 differentially expressed genes with more than a two-fold expression difference, which was the highest number of differentially expressed genes in any pairwise comparison. In contrast, soleus vs EDL and soleus vs psoas comparisons yielded 716 and 944 genes, respectively. This was surprising as we expected psoas to have an expression pattern that was closer to EDL, due to the similar Myh expression profiles between these two muscles, and the fact that they are both classified as fast-twitch muscles [8, 29]. Therefore, a principal component analysis (PCA) was carried out on the complete dataset with the marginally expressed genes removed (see methods) to determine the underlying components associated with the expression variability (Fig. 1c). The first principal component (PC1) accounted for 46% percent of the transcriptomic variability among the genes. We observed two major clusters along PC1 separating soleus from EDL and psoas. The second principal component (PC2) accounts for 27% of the expression variability among the genes. Although EDL and psoas occupy similar coordinates along PC1, they showed clear separation along PC2, where soleus clusters between EDL and psoas. The remaining principal components carried minor contributions to the observed variance among the samples (PC3 = 8.47%, PC4 = 6.59%, PC5 = 5.17%, PC6 = 4.44%, PC7 = 2.90%, PC8 = 2.84, PC9 = 1.01%).

To investigate the genes with the highest correlation to PC1 and PC2, the genes were ranked on their respective loading value, and the top 10 genes from each data set were selected (Table 1). The Pearson correlation coefficients calculated for the FPKM values of the selected genes and the respective principal components are shown in Table 1. The selected genes with the highest correlation to PC1 were related to genes in striated muscles that exhibit fiber type-specific characteristics, leading to the prominent separation of EDL and psoas from soleus along PC1. Likewise, the selected genes with the highest correlation to PC2 were associated with extracellular matrix and signaling (Table 1), and these genes do not have a direct association with a particular fiber type. The expression variability of these genes gave rise to the separate clustering of the EDL, psoas, and soleus along PC2.

**Table 1:** Genes with the highest correlation to principal components 1 and 2. The top 10 genes with the highest loading to PC1 and PC2 are shown. The Pearson correlation coefficients of FPKM values of selected genes to principal components are denoted by R^2^

Principal component	Gene symbol	Gene name	R^**2**^
PC1	Myh7	Myosin, heavy polypeptide 7, cardiac muscle, beta	0.92
	Myl2	Myosin, light polypeptide 2, regulatory, cardiac, slow	0.91
	Tnni1	Troponin I, skeletal, slow 1	0.91
	Tnnt1	Troponin T1, skeletal, slow	0.97
	Tpm3	Tropomyosin 3, gamma	0.96
	Myh6	Myosin, heavy polypeptide 6, cardiac muscle, alpha	0.89
	Atp2a2	Atpase, Ca++ transporting, cardiac muscle, slow twitch 2	0.97
	Myl3	Myosin, light polypeptide 3	0.96
	Tnnc1	Troponin C, cardiac/slow skeletal	0.94
	Strit1	Small transmembrane regulator of ion transport 1	0.87
PC2	Col11a1	Collagen, type XI, alpha 1	0.89
	Kera	Keratocan	0.92
	Wif1	Wnt inhibitory factor 1	0.91
	Tnmd	Tenomodulin	0.95
	Cilp2	Cartilage intermediate layer protein 2	0.86
	Tnc	Tenascin C	0.92
	Ecrg4	ECRG4 augurin precursor	0.87
	Lars2	Leucyl-trna synthetase, mitochondrial	−0.49
	Col1a1	Collagen, type I, alpha 1	0.80
	Col11a2	Collagen, type XI, alpha 2	0.81

Up- or down-regulated genes identified in each pairwise differential expression analysis were compared in an UpSet plot to investigate the number of unique and overlapping genes (Fig. 1d). The two largest gene subsets were associated with the psoas vs EDL pairwise comparison, showing that the expression differences observed between two fast muscles are unique to that specific pair. This is likely a characteristic of muscle specialization [3]. Even though EDL and psoas are fast muscles, the two muscles are located in different parts of the body and the functional loads imposed on them are different [20, 30]. Molecular adaptations in response to the difference in function are likely reflected by the observed gene expression differences between EDL and psoas.

### Differential expression patterns vary between muscle types based on function

**Fig. 2:**
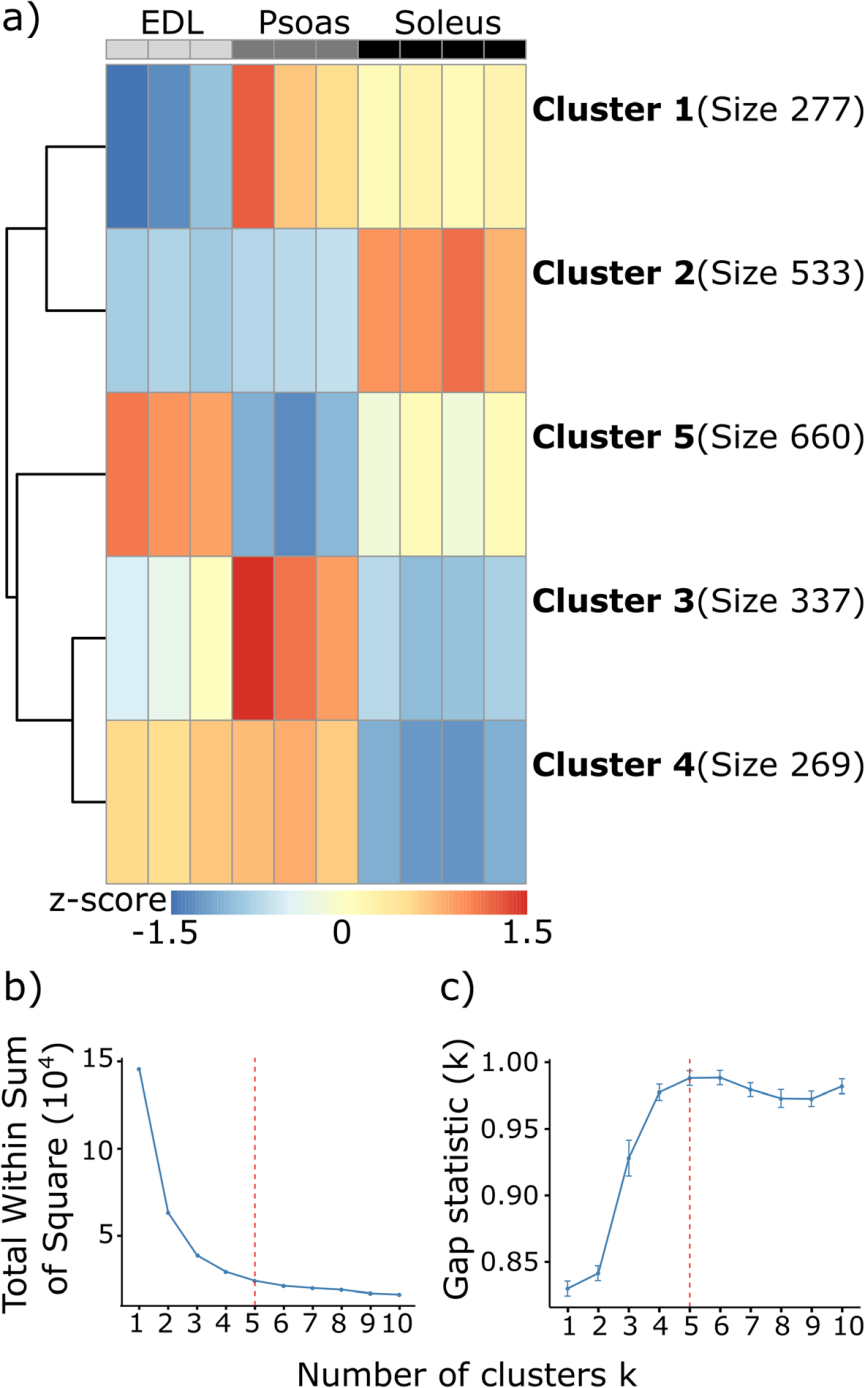
Differentially expressed genes show five distinct clusters among EDL, psoas, and soleus. **a** Z-score based hierarchal clustering of the gene subsets identified by k-means clustering of the differentially expressed genes (padj< 0.01 and fold change > 2). The optimal number of k-means clusters was determined using (**b**) elbow, and (**c**) gap statistic methods. The color intensities correspond to the average gene expression standard deviation from the mean (cluster centers). Red represents upregulation, and blue represents downregulation

Recognizing that there were clear differences in gene expression among the three muscles, we were interested in clustering differentially expressed genes by functional characteristics. Spangenburg and Booth [6] demonstrated that studying functional gene clusters that depend on common regulatory factors provides valuable insights into muscle phenotype. Using this approach, we employed k-means clustering [31–34] on the differentially expressed genes exhibiting more than a two-fold expression difference in at least one pairwise muscle comparison (Fig. 2). Based on the similarity in phenotype and myosin isoform expression patterns between psoas and EDL, we predicted that these two muscles would exhibit similar expression signatures compared to the soleus, and this pattern was discernable in the clustering analysis.

**Fig. 3:**
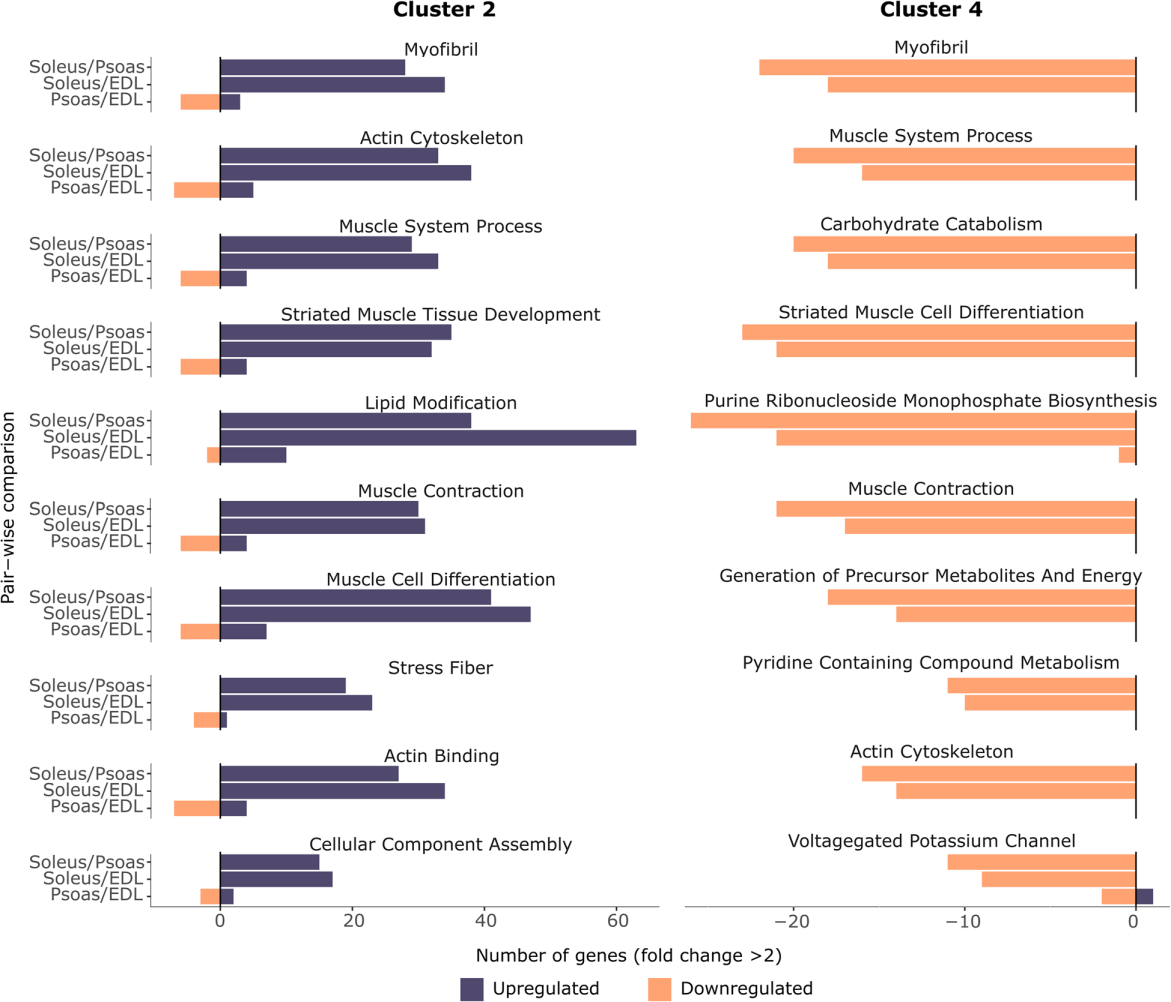
Gene clusters 2 and 4 associate with muscle structure, function, and energy metabolism and highlight the cellular processes differentiating fast and slow fiber-rich muscles. Gene ontology terms identified for gene cluster 2 and cluster 4 were summarized using REViGO online tool and the number of up and down-regulated genes (absolute log fold change > 2) associated with the top ten gene ontology terms (based on the *p*-value) were visualized in bar charts. Gene expression heatmaps associated with the bar charts for cluster 2 and cluster 4 are shown in Fig. S4 and Fig. S6 in SI

K-means clustering resulted in five gene clusters, which were plotted in a heatmap of corresponding expression differences (Fig. 2). Two primary gene expression patterns can be identified in the resulting heatmap. One is associated with the fiber type classification (Clusters 2 and 4), and the other, more specific to individual muscle characteristics (Clusters 1, 3, and 5). Among the identified gene clusters, Cluster 2 contains 533 genes that exhibit elevated expression levels in soleus relative to EDL and psoas, and Cluster 4 contained 269 genes exhibiting the opposite pattern, with soleus genes downregulated relative to EDL and psoas. The ten most significant gene ontology terms enriched from these two gene sets were associated with sarcomere structure, function, and energy metabolism (Fig. 3). The majority of the genes associated with selected gene ontology terms show concurrent up or down-regulation in soleus vs EDL, and soleus vs psoas comparisons. This was expected because both EDL and psoas are fast fiber-rich muscles [8, 29]. However, there was a smaller subset of genes associated with the selected GO terms, which showed significant expression changes in the psoas vs EDL comparison (Fig. 3, and Fig. S4 and Fig. S6 in SI).

**Fig. 4:**
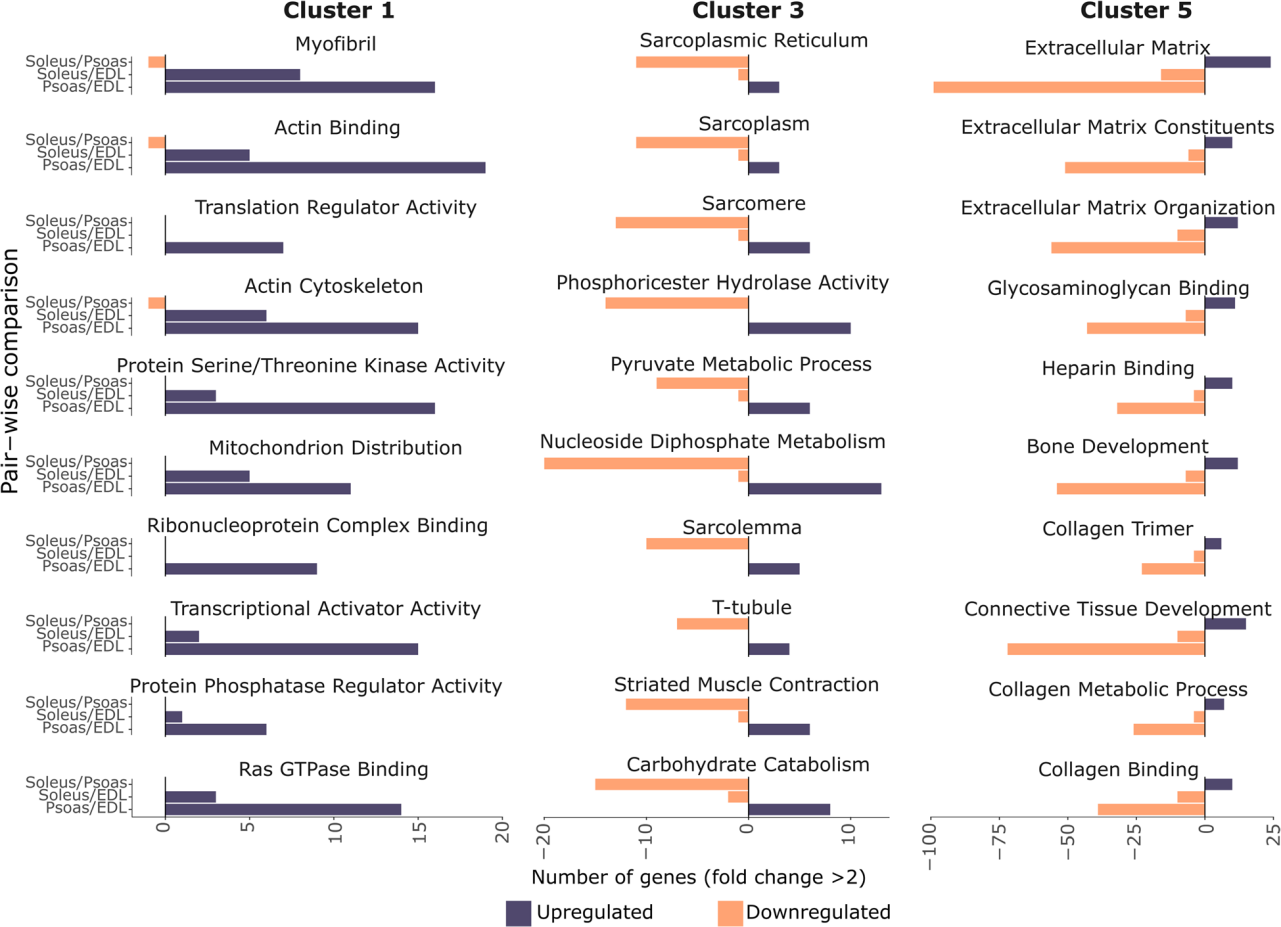
Gene clusters 1, 3, and 5 show muscle-specific characteristics of gene expression. Gene ontology terms identified for gene Clusters 1, 3, and 5 were summarized using REVIGO online tool and the number of up and down-regulated genes (absolute log fold change > 2) associated with the top ten gene ontology terms were visualized in bar charts. Gene expression heatmaps associated with the bar charts for cluster 1, cluster 3, and cluster 5 are shown in Fig. S3, Fig. S5, and Fig. S7 in SI

The remaining gene clusters showed muscle-specific expression signatures, that did not adhere to a broader fiber-type based classification of muscles. Gene clusters 1, 3, and 5 contained 277, 337, and 660 genes, respectively. The number of up or downregulated genes associated with the top 10 gene ontology terms enriched from Clusters 1, 3, and 5 are shown in Fig. 4. Even though EDL and psoas are fast fiber-rich muscles [8, 29], up or downregulated gene signatures in soleus vs EDL, and soleus vs psoas comparisons are distinctly different from one another. Furthermore, a higher number of differentially expressed genes was observed in the comparison between two fast muscles (psoas vs EDL) in association with the selected gene ontology terms for Clusters 1 and 5 (Fig. 4, and Fig. S3 and S7 in SI). The gene expression variability captured by Clusters 1, 3, and 5 emphasizes the differences between two fast muscles (EDL and psoas). They also highlight particular cases in which the expression patterns of EDL or psoas align with the slow soleus muscle. Genes in clusters 1 and 3 were associated with a wider array of ontology categories, but cluster 5 showed association with extracellular matrix components (Fig. 4, Additional files 2, 4, and 6).

The observations gathered from the gene ontology analysis can be linked back to the genes correlated with PC1 and PC2 as shown in Table 1. Clusters 2 and 4 contain genes associated with structural and functional components of muscles, that followed an expression pattern concordant with the fiber type and comparable with PC1. Clusters 1, 3, and 5 are associated with the second layer of variation among the skeletal muscles, which was also observed in the genes correlating to PC2 (Table 1). Clusters 1 and 3 highlight variations in EDL and psoas muscles associated with a multitude of structural and functional characteristics including myofibrils, mitochondrion distribution, sarcoplasm, muscle contraction, transcription, translation, and carbohydrate catabolism (Fig. 4) that are not apparent in physiological studies. Similarly, cluster 5 exhibited muscle-specific characteristics associated with the extracellular matrix (ECM) components that did not directly follow fiber-type (Fig. 4 and Fig. S5). These observations agree with the findings reported by Prado et al. [35], who observed a higher contribution of ECM to passive stiffness in both soleus and EDL relative to the psoas. Generally, slow oxidative muscles like soleus contain a more extensive collagen fiber network than fast muscles [35]. However, this is not universally the case as EDL has more collagen than psoas [35, 36]. Taken together, it is hard to establish a correlation between fiber type and ECM thickness among EDL, psoas, and soleus, which is reflected in our transcriptomic data.

### Slow fiber specific isoforms play a central role in developing slow muscle characteristics

**Fig. 5:**
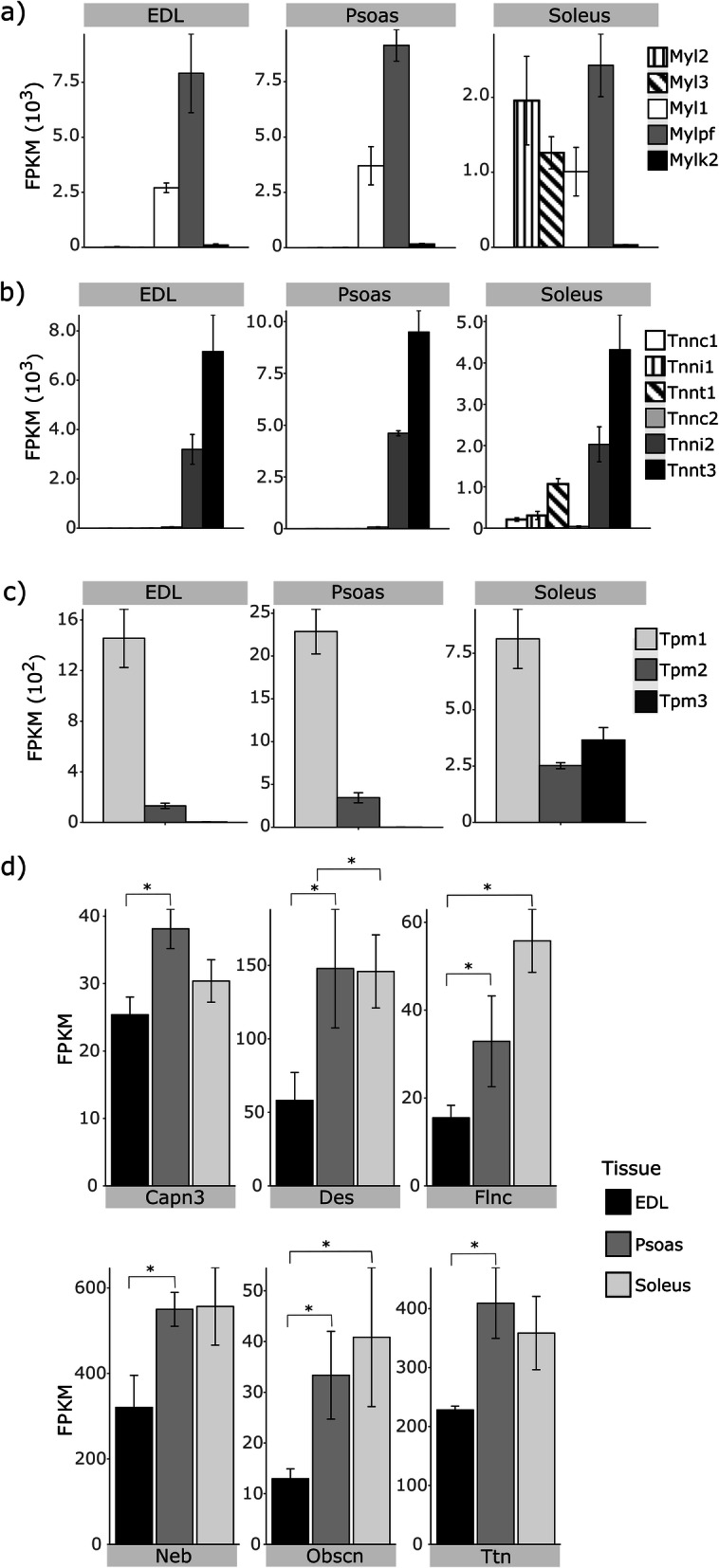
Isoforms associated with fast-twitch fibers are expressed in all three muscles. Expression levels of (**a**) Myosin light chains, (**b**) troponin, and (**c**) tropomyosin isoforms in EDL, psoas, and soleus. Fast-twitch myosin light chain isoforms (Myl1, Mylk2, and Mylpf), troponin isoforms (Tnnc2, Tnni2, and Tnnt3), and tropomyosin 1 (Tpm1) are the expressed in all three muscles, but the expression levels are notably higher in EDL and psoas. At least 97-fold higher expression of slow myosin light chain isoforms Myl2 and Myl3, 137-fold expression upregulation of slow troponin isoforms Tnnc1, Tnni1, and Tnnt1, and 31-fold expression upregulation in Tpm3 are observed in soleus compared to EDL and psoas. Tpm2 is preferentially expressed in slow muscles, but 2.4 fold up-regulated was observed in psoas compared to EDL (*p-adj* = 6.37e^− 10^). **d** Genes coding for proteins that interact at the Z-disk are downregulated in EDL. The calpain 3 (Capn3), desmin (Des), filamin-C (Flnc), nebulin (Neb), obscurin (Obscn), and titin (Ttn) follow a similar expression signature, where all six genes are significantly downregulated in EDL compared to the psoas. ^*^ *p-adj* < 0.01. Adjusted *p* values were calculated using DESeq2

In addition to the global gene expression differences observed in the study, specific sets of genes, such as the myosins, troponins, and tropomyosins are of particular interest as these contain sets of slow and fast-fiber specific isoforms that are coded by different genes. Therefore, we looked at the gene expression levels related to these thin and thick filament proteins in more detail. As shown earlier, EDL and psoas predominantly express a fast-twitch isoform of the myosin heavy chain, while soleus expresses a mixture of the four main myosin heavy chain isoforms (Fig. 1a). Interestingly, when we compared the gene expression levels of the myosin light chains (Myl) (Fig. 5a), troponins (Fig. 5b), and tropomyosins (Fig. 5c), we observed a unique set of features. The slow-fiber related isoforms are expressed only in soleus, while the fast isoforms are expressed in all three muscles. Slow fiber associated Myl2 and Myl3 showed high expression levels in soleus, but extremely low levels in EDL and psoas. In contrast, fast-fiber related Myl1, Mylpf, and Mylk2 exhibit comparatively high expression levels in all three muscles, though EDL and psoas had higher levels than the soleus. Similar gene expression trends were observed in troponin transcripts, where slow isoforms - Tnnc1, Tnni1, and Tnnt1 were markedly high in soleus, while fast isoforms - Tnnc2, Tnni2, and Tnnt3 were found in all three muscles. In tropomyosin, Tpm1 (prominent in fast muscles [37]) is expressed in all three muscles, though higher expression levels were observed in EDL and psoas. Tpm2 (prominent in slow muscles [37]) showed elevated levels in the psoas compared to EDL (psoas vs EDL *p-adj = 6.37e-10*). Furthermore, Tpm3 transcript levels were markedly high in soleus compared to EDL or psoas (soleus vs EDL *p-adj* = 8.23e-75, soleus vs psoas *p-adj* = 4.56e-112). This unique pattern of slow isoform expression among different muscles suggests a significant role played by the expression ratio of fast and slow isoforms in tuning the phenotype of different skeletal muscles.

### Genes associated with sarcomere Z-disk display a unique expression signature in EDL

The Z-disk is a dense network of proteins anchoring thin filaments from opposing sarcomeres to actinins, titin, and many other structural and signaling proteins [38, 39]. We observed a unique expression pattern in the genes coding for the proteins that assemble in this region, in which EDL exhibits lower expression levels compared to the psoas. We have studied six genes that are associated with the z-disk [40, 41] (Fig. 5d). Titin shows 1.7-fold expression upregulation in psoas vs EDL (*p-adj = 0.0005*). Nebulin (Neb), obscurin (Obscn), calpain3 (Capn3), desmin (Des), and filamin-C (Flnc) follow a similar expression signature to titin. These observations suggest possible structural or functional variability at the Z-disk of the fast muscle sarcomere [42, 43], which may lead them to respond to cellular perturbations differently.

## Discussion

Skeletal muscle is a complex network of proteins that work together to generate the forces necessary for movement. The diversity of muscle function spans from involuntary and rhythmic movements of respiratory muscles to the fine contractions of extraocular muscles to limb muscles, which have variable endurance capacities [3]. The same general architecture is used to accomplish these different tasks by tuning isoform expression levels of certain critical proteins [2, 3]. Contractile properties such as muscle shortening velocity, twitch duration, and endurance capacity, as well as metabolic properties which control the rate of ATP synthesis and hydrolysis [44], play a central role in skeletal muscle diversity. These characteristics depend on which isoforms, or combination of isoforms, are expressed in a particular muscle fiber. One of the primary methods for identifying fiber type is the determination of the expression profile of fast and slow myosin heavy chain isoforms. This approach provides general insights, but many muscle-specific differences are not captured through this approach [3]. Within both the fast and slow fiber classifications, there is tremendous variability in many important gene families, which creates subtle distinctions between the various muscles within these two broad categories. To start to understand this variability, we used RNA sequencing to explore gene expression differences in EDL, psoas, and soleus muscles. Our data shows that there is a rich diversity of gene expression beyond myosin isoforms that defines the characteristics of any muscle fiber, and this diversity results in the range of muscle phenotypes that are known to exist.

### Transcription of muscle-specific genes does not strictly adhere to fast and slow fiber types

In the current study, we show that the fast vs. slow-twitch classification of muscles can be explained by the most prominent transcriptomic changes among the muscles, which agrees with the traditional perspective. This claim was supported by the major clustering along the first principal component (PC1) in the principal component analysis, and the correlation of muscle-specific genes to PC1. This was also reflected in gene Clusters 2 and 4, identified from the k-means cluster analysis, showing opposite expression patterns, when the slow muscle (soleus) was compared to the fast muscles (EDL and psoas). Clusters 2 and 4 were primarily associated with muscle structure, contraction, and energy metabolism, which are the predominant cellular characteristics differentiating fast muscles from slow muscles [2]. This can be considered as the most prominent layer of transcriptomic differences among the skeletal muscles.

Previous studies have highlighted the importance of considering multiple functional gene groupings in addition to myosin heavy chain to characterize different skeletal muscles [3, 6]. Our study supports this conclusion since we identified a second layer of transcriptomic differences within our data set. This was highlighted by EDL, psoas, and soleus forming distinctly separated clusters along the second principal component (PC2) in the principal component analysis, which were not correlated with the fast vs. slow-twitch classification of muscles. Genes showing the highest correlation to PC2 were associated with extracellular matrix (ECM) and signaling. ECM has been shown to be associated with muscle-specific behavior in previous physiological studies [35] and this conclusion is supported by gene Cluster 5, which showed a strong association with the extracellular matrix.

Gene clusters identified from the study also revealed an additional layer of transcriptomic differences between EDL, psoas, and soleus. Underneath the first and the second layers of transcriptomic differences discussed previously, the gene expression variability captured by Clusters 1 and 3 highlight a wide array of gene ontologies associated with the differences between fast muscle (EDL and psoas). Both gene clusters were associated with a multitude of structural and functional characteristics including myofibrils, mitochondrion distribution, sarcoplasm, muscle contraction, transcription, translation, and carbohydrate catabolism (Fig. 4) that are not easily identified in physiological studies.

These observations further highlight the value of considering transcriptomic diversity when trying to understand the molecular underpinnings of specific muscle functions. Genes associated with subtle processes, such as transcription, translation, carbohydrate metabolism, and muscle adaptation, are not as closely associated with fiber type. These molecular processes are more easily overlooked or hard to measure, making them less apparent in most physiological measurements. Our results highlight that muscle fibers are multi-faceted structures and that differences exist among muscles with diverse contractile properties. We predict that similar profiles could be developed for most skeletal muscles, providing new insights into the function of various muscles.

Gene expression differences identified from this study may help explain why EDL, psoas, and soleus muscles are specifically targeted or spared in certain types of myopathies [3, 20]. For example, the response severity of EDL and psoas to muscular dystrophy with myositis is different from one another, even though the wildtype muscles contain similar myosin heavy chain profiles [45]. By utilizing transcriptomic level information, the skeletal muscles can be compared with respect to their response pattern to a given muscle disease [46]. The transcriptomic changes introduce an additional layer of information, which helps to differentiate muscles even when their fiber type composition is similar. These transcriptomic signatures can be used to identify comparable characteristics among diverse sets of muscles. In such cases, variable muscle response to myopathic conditions could be associated with shared transcriptomic behaviors.

### The expression ratio between slow and fast fiber specific isoforms act as a determining factor of muscle function

The development of fast and slow-twitch phenotypes is associated with the expression of Myh4 and Myh7 isoforms, which act as the fingerprints of those respective muscle phenotypes [3, 47]. Both EDL and psoas do not express Myh7, and the expression of Myh4 in soleus is about 5% of the total myosin content. However, fast-twitch muscles still express a small percentage of slow myosin isoforms, and slow-twitch muscles express fast myosin isoforms. Interestingly, we have observed that the level of fast isoform expression in the slow muscle soleus is greater than the expression level of slow isoform in fast muscles EDL and psoas. This could be a function of the fiber composition of soleus. Expression of both slow- and fast-twitch related isoforms of myosin light chain (Myl), troponin (Tnnc, Tnni, Tnnt), and tropomyosin (Tpm) in skeletal muscles have previously observed in other studies as well [48–51]. Expression of fast Myl isoforms was observed in slow-twitch muscles in both humans and rats, while slow Myl isoform expression was reported in the rat fast-twitch EDL and plantaris muscles [49, 51]. In this study, we observed that the fast and slow transcript levels for Myl in soleus are nearly identical. Myl is associated with shortening velocity, but soleus phenotypically behaves as a slow-twitch muscle, even though there are equivalent transcript levels of both fast and slow Myl isoforms. This suggests the possibility that the slow isoform playing a larger role in determining muscle phenotype when equivalent expression levels exist.

This possibility is further highlighted by tropomyosin expression patterns. The expression ratio between slow to fast tropomyosin isoforms is associated with fiber type, with a more prominent expression of Tpm3 in slow muscles [52]. In our studies, the observed ratio of slow muscle associated tropomyosin (Tpm2 and Tpm3) to fast muscle associated tropomyosin (Tpm1) expression in psoas is significantly greater than that of EDL. This could be associated with other transcriptomic variabilities observed in fast muscles, as tropomyosin plays a critical role in controlling Ca^2+^ sensitivity [53]. The co-expression of both Tpm1 and Tpm2 in psoas has previously been observed in a rabbit mRNA study [50]. While psoas is typically characterized as a fast-twitch muscle, it does exhibit some slow-twitch characteristics in certain experiments [54], which could be a function (at least in part) of expressed tropomyosin isoforms ratios. These observations provide an example of how tuning isoform expression could modify muscle physiology.

In addition to the contractile machinery of the sarcomere, other structural proteins exhibit variation in expression between EDL and psoas. Titin, calpain-3, obscurin, desmin, nebulin, and filamin-C are all associated with the Z-disc and they exhibit clear expression differences between EDL and psoas. No significant expression difference between psoas and soleus were observed in these genes at the 99% significant level. Expression differences between psoas and EDL have been previously observed in desmin [55], and titin [56], but differences in the transcript levels for these proteins have not been previously correlated with differences among fast muscles.

The Z-disk periodicity of fast muscles is lower than that of slow muscles, so that slow muscles have a thicker Z-band compared to fast muscles [57, 58]. The Z-band thickness depends on the number of Z-repeats of titin, which interacts with α-actinin. The psoas tends to express a very short Z-repeat domain in titin, where titin length [59], and the Z-repeat expression [58] vary as a function of muscle type (psoas< EDL < soleus). It was surprising to observe similar expression profiles in psoas and soleus associated with Z-disk proteins even though they have distinct Z-disk periodicity. There is a clear difference between the titin-based passive force generation in psoas compared to EDL [35]. As the Z-disk is a signaling hub of the sarcomere and is also involved in force transmission between neighboring sarcomeres, it is reasonable to propose that the expression patterns we observed in Z-disk proteins are associated with the different passive force profiles of EDL and psoas. It is important to note that transcriptomic differences do not always correlate with protein dynamics inside a cell. However, transcription is one of the key response mechanisms related to changing cellular needs and provides a guide for future investigations. Identifying these subtle but distinct changes at transcription level between muscles with similar fiber compositions can provide valuable insights into why muscles of the same overall fiber type may respond differently to genetic or environmental perturbations.

## Conclusions

Skeletal muscles are traditionally categorized as fast-twitch or slow-twitch based on fiber composition, which is often assessed using myosin heavy chain expression profiles. However, more than half of the transcriptome undergoes significant expression changes [3], which can be used to differentiate skeletal muscles from each other. The results presented in this study support the traditional view of muscle fiber types, but also demonstrate the importance of using a more comprehensive view of gene expression differences to understand subtle differences in function among muscles. There are significant gene expression differences between two fast muscles EDL and psoas, associated with the myofibril, transcription, translation, actin cytoskeleton, sarcoplasmic reticulum, energy metabolism, and extracellular matrix. These observations highlight the importance of considering the expression ratios of slow and fast isoforms of a variety of muscle-related protein-coding genes to differentiate muscles with similar fiber type composition and also to correlate skeletal muscles regardless of the general muscle fiber type classification. The gene sets undergoing similar expression regulation between muscles could help to understand the complex phenotypes of various myopathies and possibly identify hitherto unknown players associated with muscle diseases.

## Methods

### Sample preparation

The mouse colony was established at the animal care facility of Northern Arizona University, Flagstaff, AZ, USA, using heterozygous B6C3Fe a/a-Ttn^mdm^/J mice obtained from The Jackson Laboratory (Bar Harbor, ME, USA). Mice were fed ad libitum and maintained under light: dark 12 h:12 h cycle in a temperature-controlled facility. Mouse muscle samples collected from 32 to 54-day old homozygous wild-type mice were used for the study (Table S1). The experimental protocol and the use of animals were approved by the Institutional Animal Care and Use Committee at NAU (Reference number:18–002).

Three biological replicates for EDL and psoas muscles and four biological replicates for soleus muscle were extracted from a total of 6 mice. Mice were sacrificed under 0.5 ml of isoflurane gas in a euthanization chamber and were conscious prior to the treatment. Extracted muscle tissues were stored in RNAlater™ stabilization solution (ThermoFisher Scientific) at − 80 °C before RNA extraction. The tissues collected for this study were harvested from mice subjected to another study to minimize the number of euthanized mice. The six mice used in the study were determined by the availability of the required muscles based on the other experimental study conducted with the mice. Power analysis for the transcriptomic study was carried out using Scotty - Power Analysis for RNA Seq Experiments tool (http://scotty.genetics.utah.edu/) with a 70% alignment rate and 20% or above gene detection while keeping other parameters at their default values.

### RNA extraction and next-generation sequencing

Total RNA was extracted from the collected muscle tissues using Qiagen fibrous tissue total RNA extraction mini kit. The concentration and quality of RNA were measured using the Qubit RNA Broad-Range assay and Agilent 2100 Bioanalyzer RNA 6000 Nano assay. Samples with RNA integrity value over seven and a concentration that was greater than 20 ng/μl were selected for cDNA library preparation using an Illumina TruSeq Stranded Total RNA Library Prep kit with Ribo-Zero Globin treatment. The cDNA libraries were quantified using KAPA Library Quantification qPCR Kit for Illumina sequencing platforms. The library fragment sizes were determined using an Agilent bioanalyzer 2100 High Sensitivity dsDNA quantification assay. The mean library sizes of the samples were between 256 and 295 bp (Table S1). The cDNA libraries were sequenced and 2 × 75 bp reads were generated with an Illumina NextSeq 500 high throughput sequencer using the sequencing guidelines suggested by Illumina, over three sequencing runs. The sequenced library coverage varies between 12 and 82 million reads with a median of 25 million (Table S1).

### Raw data processing

The fastq files containing the short reads generated from RNA sequencing were processed before analysis. The quality of the short-read files was assessed using the FastQC quality control tool (http://www.bioinformatics.babraham.ac.uk/projects/fastqc/) for high throughput sequence data. Fastq files with a per base quality score below 20 were preprocessed using the sliding window quality filtering (window size 4) in Trimmomatic v0.32 [60]. After filtering, only the paired-end reads collected from the read-trimming were used for downstream data analysis (Table S1). Fastq files containing reads with more than 20 per base quality score were used without filtering. Adaptor trimming was performed while converting initial BCL data to fastq files before receiving the data files from the sequencing center. No adaptor contamination was detected in the FASTQC analysis for the samples used in the study.

### Data alignment

The preprocessed fastq files were aligned to the mouse reference genome as follows. The insert sizes between paired-end reads were calculated using the Galaxy web platform [61]. A subset of 250,000 reads from each sample was aligned to the built-in reference mouse genome (mm10) using the default settings in the BWA-aln short read alignment tool [62]. The CollectInsertSizeMetrics Picard tool (http://broadinstitute.github.io/picard/) was used to generate the alignment statistics. The calculated average insert sizes and standard deviations of each sample were then used to generate complete read alignments using Tophat v2.1.1 [63], using the *Mus musculus* GRCm38.p4 genome annotation (see alignment details in Table S1).

### Differential gene expression analysis

Statistical analysis of generated read alignments was performed using R_3.6.1. Gene-wise read counts of the Tophat alignments were generated using the R libraries GenomicFeatures v1.34.3 [64] and GenomicAlignments v1.18.1. To reduce the noise in the data and increase the precision of data analysis [65] the marginally expressed genes were filtered out. Genes were identified as marginally expressed if a gene carries less than three samples with a minimum read count of 10 [65] among all the samples in the data set and these genes were removed from the dataset before downstream data analysis. This filter resulted in a gene subset of 16,112 genes out of the 46,078 genes in the reference genome. A heatmap of this gene set is shown in Fig. S2 in SI. To determine whether the variable read coverages affects the correlation among replicates, a distance matrix was calculated prior to differential gene expression analysis using variance stabilized normalized (DESeq2::vst) and sequencing batch effect corrected (limma:: removeBatchEffect) [66] gene expression levels of selected 16,112 genes using R dist function (Fig. S1). Replicates of each muscle created unique clusters, while two fast muscles (EDL and psoas) branch discretely from slow soleus.

To compare the transcriptomic profiles of EDL, psoas, and soleus, the differential gene expression analysis was performed using the R library DESeq2 v1.22 [67, 68]. In the DESeq2 protocol, the RNA-Seq data get fitted with a generalized linear negative binomial distribution model to calculate subtle changes in gene expression. Then to assess the statistical significance of differential gene expression DESeq2 utilizes a Wald test as the dataset has been modeled as a binomial distribution. The *P*-values calculated were further adjusted using the Benjamini-Hochberg multiple testing procedure [69] in the subsequent DESeq2 protocol steps. After that, the significantly differentially expressed genes in the pair-wise comparisons among EDL, psoas, and soleus were identified using adjusted *p*-value cut-off 0.01 (at the 99% significance level) and fold change cutoff of 2. A higher significance level cut-off was used to avoid possible selection bias due to the small sample size of the data set.

### Gene ontology analysis

Gene ontology (GO) analysis was performed to determine the cellular processes associated with the differentially expressed genes using ClusterProfiler_3.10.1 [70] against the org.Mm.eg.db v3.7.0 - Bioconductor genome-wide annotation for the mouse [71], with a q-value cut-off of 0.05. The semantically similar terms of the identified GO terms were removed using SimRel semantic clustering in the REViGO [72] online tool with reference to the in-built *Mus musculus* Jan 2017 database release by the GO Consortium. The GO id list and the respective adjusted *p*-values generated from clusterProfiler R library were used as the input to REViGO and GO term summaries were generated with 0.5 allowed similarity. The top 10 gene ontology terms enriched from each gene cluster were selected by sorting the REViGO selected GO terms by their respective log10 p-value in the resulting list (Additional files 2, 3, 4, 5 and 6). The number of up- or down-regulated genes associated with selected GO terms were identified using the complete gene set associated with all the semantically similar go terms represented by the chosen parent terms by REViGO to create the bar charts.

### Data visualization and graphical output generation

Variance stabilizing transformed and sequencing batch effect corrected, gene expression levels of the differentially expressed genes were used to create the heatmaps. The Z-scores calculated for the differentially expressed genes using this dataset were used for k-means clustering. The optimal number of gene clusters were determined [33] using elbow and gap statistics methods using R function NbClust::fviz_nbclust (https://sites.google.com/site/malikacharrad/research/nbclust-package) keeping other parameters at default values. The R library Pheatmap_1.0.12 (https://cran.r-project.org/web/packages/pheatmap/index.html) was used for clustering and to generate the heatmap of gene clusters (Fig. 2). Complete lists of genes in each cluster are included in the Additional files 2, 3, 4, 5 and 6.

The UpSet plot was created using R library UpSetR_1.4.0 [73]. The bar charts were generated using the R library ggplot2_ 3.3.2 (https://ggplot2.tidyverse.org/).

Gene expression levels were converted to Fragments Per Kilobase Million (FPKM) using DESeq2::fpkm function keeping the parameters at default values, where the gene lengths were determined with the union of all GRanges assigned to a given gene in the data object. (https://www.rdocumentation.org/packages/DESeq2/versions/1.12.3/topics/fpkm). Calculated FPKM values for the differentially expressed genes are in Additional file 7.

Gene subset selected by filtering marginally expressed genes were used to carry out the principal component analysis (PCA). Their gene expression values were subjected to variance stabilization transformation, and batch effect correction as described above. R prcomp function with default parameters was used to carry out PCA with the transformed and corrected gene expression values. To calculate the Pearson correlation coefficients between PCs and gene expression, FPKM values of the same gene set, and the PC scores (coordinates of the individual observations on the principal components calculated with R prcomp function) were used as the inputs for R cor function, while keeping other parameters at default values.

## Supplementary Information

**Additional file 1.** Additional figures and tables supporting the main text of the manuscript. Referred as SI in the main text.

**Additional file 2.** Gene ontologies and differential gene expression analysis results associated with the genes in cluster 1 identified from k-means clustering of differentially expressed genes.

**Additional file 3.** Gene ontologies and differential gene expression analysis results associated with the genes in cluster 2 identified from k-means clustering of differentially expressed genes.

**Additional file 4.** Gene ontologies and differential gene expression analysis results associated with the genes in cluster 3 identified from k-means clustering of differentially expressed genes.

**Additional file 5.** Gene ontologies and differential gene expression analysis results associated with the genes in cluster 4 identified from k-means clustering of differentially expressed genes.

**Additional file 6.** Gene ontologies and differential gene expression analysis results associated with the genes in cluster 5 identified from k-means clustering of differentially expressed genes.

**Additional file 7.** FPKM values calculated for the differentially expressed genes identified from pairwise comparisons among EDL, psoas, and soleus are shown in the table.

## Data Availability

The raw datasets used and/or analyzed in the current study have been deposited in Gene Expression Omnibus (GEO accession number GSE158283).

## Abbreviations

Atp2a2: Sarcoplasmic/endoplasmic reticulum calcium atpase 2
Ca2 +: Calcium ion.
Capn3: Calpain3
Cilp2: Cartilage intermediate layer protein 2
Col11a1: Collagen, type XI, alpha 1
Col11a2: Collagen, type XI, alpha 2
Col1a1: Collagen, type I, alpha 1
Des: Desmin
DMD: Duchenne muscular dystrophy
ECM: Extracellular matrix
Ecrg4: ECRG4 augurin precursor
EDL: Extensor digitorum longus
Flnc: Filamin C
GO: Gene ontology
Kera: Keratocan
LARS2: Leucyl-trna synthetase, mitochondrial
Myh: Myosin heavy chain
Myh-1: Myosin heavy chain type 1, protein isoform
Myh1: Myosin, heavy polypeptide 1, skeletal muscle, adult
Myh2: Myosin, heavy polypeptide 2, skeletal muscle, adult
Myh-2A: Myosin Heavy Chain type-2A, protein isoform
Myh-2B: Myosin Heavy Chain type-2B, protein isoform
Myh-2X: Myosin Heavy Chain type-2X, protein isoform
Myh4: Myosin, heavy polypeptide 4, skeletal muscle
Myh6: Myosin, heavy polypeptide 6, cardiac muscle, alpha
Myh7: Myosin, heavy polypeptide 7, cardiac muscle, beta
Myl1: Myosin, light polypeptide 1
Myl2: Myosin, light polypeptide 2, regulatory, cardiac, slow
Myl3: Myosin, light polypeptide 3
Mylk2: Myosin, light polypeptide kinase
Myl: Myosin light chain
Mylpf: Myosin light chain, phosphorylatable, fast skeletal muscle
Neb: Nebulin
Obscn: Obscurin
PC: Principal component
PCA: Principal component analysis
RNA-Seq: Ribonucleic acid sequencing
SDS-PAGE: Sodium dodecyl sulfate polyacrylamide gel electrophoresis
Strit1: Small transmembrane regulator of ion transport 1
Tnc: Tenascin C
Tnmd: Tenomodulin
Tnnc1: Troponin C, slow skeletal and cardiac muscles
Tnnc2: Troponin C, fast skeletal muscle
Tnni1: Troponin I, slow skeletal muscle
Tnni2: Troponin I, fast skeletal muscle
Tnnt1: Troponin T, slow skeletal muscle
Tnnt3: Troponin T, fast skeletal muscle
Tpm1: Tropomyosin alpha-1 chain
Tpm2: Tropomyosin beta chain
Tpm3: Tropomyosin alpha-3 chain
Wif1: Wnt inhibitory factor 1
