# Supplementary material for: Comparative analysis of the transcriptomes of EDL, psoas, and soleus muscles from mice
Source: BMC Genomics. 2020 Nov 19;21:808. doi: 10.1186/s12864-020-07225-2 (PMC7678079; doi:10.1186/s12864-020-07225-2)
Supplement: Supplementary file 1 — Additional file 1. Additional figures and tables supporting the main text of the manuscript. Referred as SI in the main text. [file 12864_2020_7225_MOESM1_ESM.zip › Additional File 1.docx]

**Supplementary information**

Table S1: RNA-Seq read alignment summary for the samples used in the study

|  | | EDL_R1 | EDL_R2 | EDL_R3 | Psoas_R1 | Psoas_R2 | Psoas_R3 | Soleus_R1 | Soleus_R2 | Soleus_R3 | Soleus_R4 |
| --- | --- | --- | --- | --- | --- | --- | --- | --- | --- | --- | --- |
| Age (days) | | 42 | 54 | 44 | 42 | 54 | 44 | 42 | 44 | 32 | 44 |
| RNA integrity number (RIN) | | 8.4 | 8.4 | 8.5 | 7.3 | 8.1 | 8 | 8.2 | 8.1 | 7.6 | 8.1 |
| Library size | | 274 | 264 | 278 | 256 | 271 | 277 | 258 | 295 | 265 | 276 |
| Left reads: | Initial number of reads | 87185432 | 57368915 | 15186094 | 1E+08 | 45937372 | 13847980 | 22022374 | 28236087 | 12437526 | 15940351 |
|  | Minimum per base quality score (10th percentile) | 14 | 14 | 27 | 14 | 14 | 32 | 32 | 32 | 32 | 32 |
|  | Percent filtered in (%) | 81.38 | 80.83 | N/A | 81.86 | 80.26 | N/A | N/A | N/A | N/A | N/A |
|  | Alignment input | 70950705 | 46369117 | 15186094 | 81953371 | 36870545 | 13847980 | 22022374 | 28236087 | 12437526 | 15940351 |
|  | Mapped | 55572350 | 44539661 | 14774183 | 62419592 | 28077507 | 13534841 | 17953627 | 27374091 | 12109861 | 15593067 |
|  | Multiple alignments | 18.5% | 19.1% | 9.4% | 18.7% | 15.9% | 9.9% | 15.2% | 9.4% | 12.9% | 9.4% |
|  | Percent mapped | 78.3% | 96.1% | 97.3% | 76.2% | 76.2% | 97.7% | 81.5% | 96.9% | 97.4% | 97.8% |
|  | | | | | | | | | | | |
| Right reads: | Initial number of reads | 87185432 | 57368915 | 15186094 | 1E+08 | 45937372 | 13847980 | 22022374 | 28236087 | 12437526 | 15940351 |
|  | Minimum per base quality score (10th percentile) | 14 | 14 | 27 | 14 | 14 | 27 | 27 | 27 | 27 | 27 |
|  | Percent filtered in (%) | 81.38 | 80.83 | N/A | 81.86 | 80.26 | N/A | N/A | N/A | N/A | N/A |
|  | Alignment input | 70950705 | 46369117 | 15186094 | 81953371 | 36870545 | 13847980 | 22022374 | 28236087 | 12437526 | 15940351 |
|  | Mapped | 56026587 | 44570183 | 14597762 | 62738333 | 28269426 | 13259768 | 17647947 | 26044316 | 11953398 | 15344925 |
|  | Multiple alignments | 18.4% | 19.2% | 9.4% | 18.7% | 8.2% | 9.9% | 15.1% | 9.3% | 12.8% | 9.4% |
|  | Percent mapped | 79.0% | 96.1% | 96.1% | 76.2% | 76.7% | 95.8% | 80.1% | 92.2% | 96.1% | 96.3% |

**Table S2: Myosin heavy chain gene expression levels obtained from the current study agree with the previous studies.** Myh isoform expression in mouse EDL, psoas, and soleus from the present study compared to the proteomics and transcriptomics data from previous studies.

|  | This work | | | | Literature | | | | | P-value | | | |
| --- | --- | --- | --- | --- | --- | --- | --- | --- | --- | --- | --- | --- | --- |
|  | Myh7 | Myh2 | Myh1 | Myh4 | Myh7 | Myh2 | Myh1 | Myh4 | Ref | Myh7 | Myh2 | Myh1 | Myh4 |
|  | Type 1 | Type 2A | Type 2X | Type 2B | Type 1 | Type 2A | Type 2X | Type 2B |  | Type 1 | Type 2A | Type 2X | Type 2B |
| EDL | 0.06%±0.05 | 4% ±1.17 | 17% ±2.38 | 79% ±3.39 | 1% | - | 36% | 63% | [24] | 0.3364 | 0.8697 | 0.1424 | 0.1899 |
|  |  |  |  |  | - | 10.2% | 23.7% | 55.7% | [8] |  |  |  |  |
|  |  |  |  |  | 0% | 3% | 25% | 72% | [13] |  |  |  |  |
|  |  |  |  |  | 4% | 3% | 30% | 63% | [29] |  |  |  |  |
|  |  |  |  |  | 0% | 2% | 27% | 71% |  |  |  |  |  |
|  |  |  |  |  | - | 1% | 10% | 89% | [3]* |  |  |  |  |
| Psoas | 0.006% ± 0.003 | 5% ±1.92 | 16% ±4.68 | 79% ±6.61 | - | 15% | 17% | 68% | [26] | 0.3779 | 0.2117 | 0.7862 | 0.2519 |
|  |  |  |  |  | 2% | 32% | - | 65% | [27] |  |  |  |  |
|  |  |  |  |  | 0% | 11% | 21% | 68% | [29] |  |  |  |  |
|  |  |  |  |  | 0% | 3% | 14% | 83% |  |  |  |  |  |
| Soleus | 30% ±8.75 | 39% ±4.31 | 26% ±9.72 | 5% ±2.78 | 37% | - | 63%ᶤ |  | [24] | 0.5106 | 0.0525 | 0.0643 | 0.2008 |
|  |  |  |  |  | 30.6% | 49.1% | 11.8% | 3.1% | [8] |  |  |  |  |
|  |  |  |  |  | 35% | 53% | 12% | 0% | [13] |  |  |  |  |
|  |  |  |  |  | 48% | 37% | 11% | 0% | [29] |  |  |  |  |
|  |  |  |  |  | 28% | 47% | 17% | 7% |  |  |  |  |  |
|  |  |  |  |  | 26% | 52% | 20% | 2% | [3]* |  |  |  |  |

The third column contains protein expression data gathered from the literature except the rows containing * which shows transcriptomics data by Terry et al [3]. ᶤ Excluded from the T-test, as the value corresponds to the total percentage of type 2 myosin heavy chain isoforms.

**
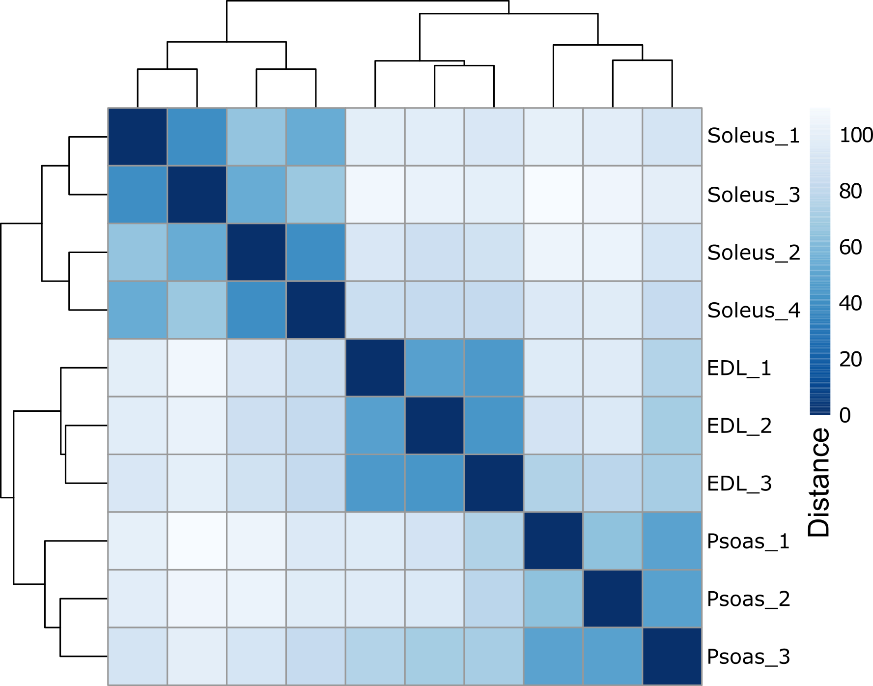
**

**Figure S1:** Sample to sample distance among the replicates of EDL, psoas and soleus. Hierarchical clustering of the samples based on the Euclidian distance matrix calculated for the batch effect corrected, variance stabilized normalized counts (DESeq2::vst) of the 16112 genes used for the differential gene expression analysis.


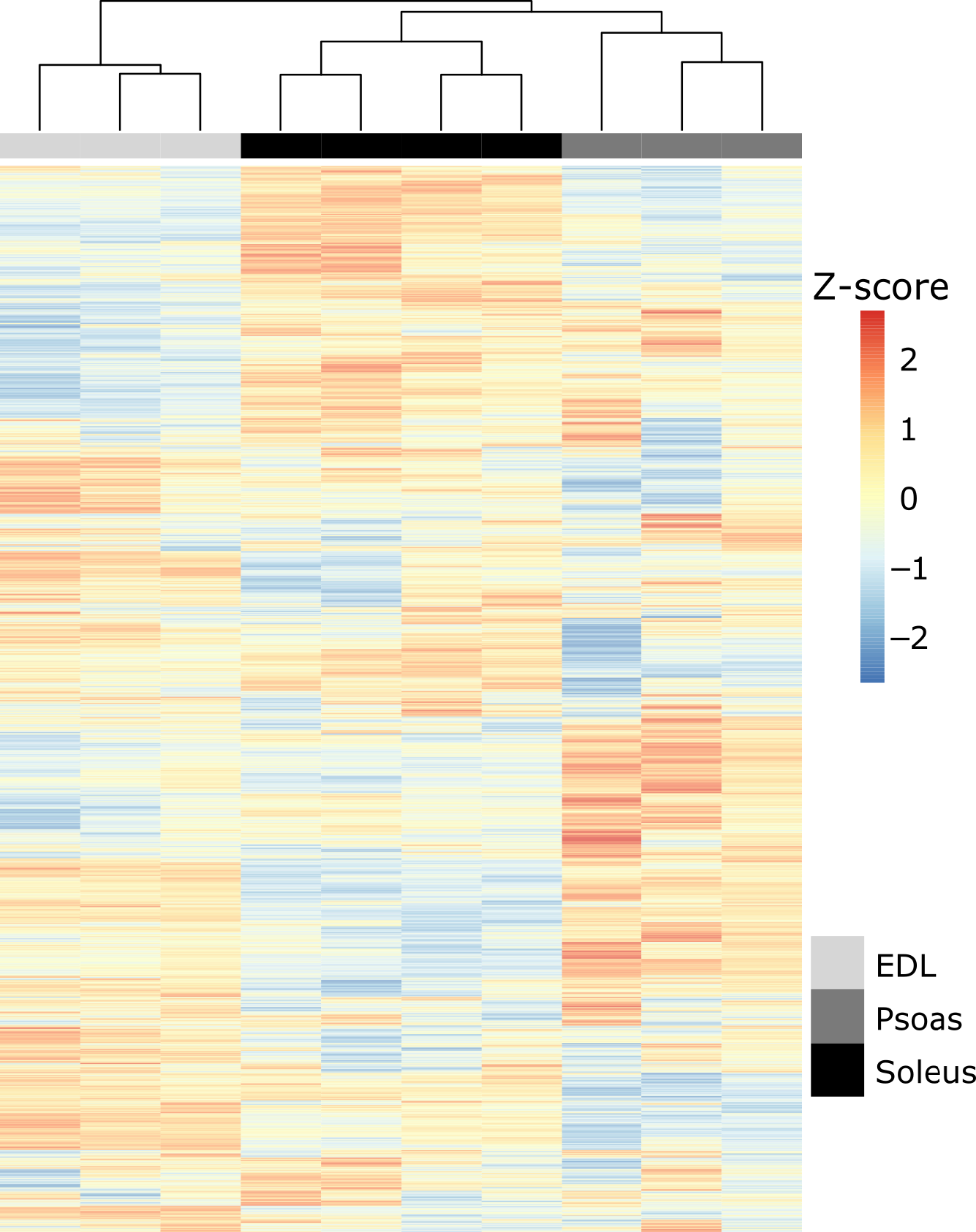


**Figure S2:** Heatmap showing Z-score based hierarchal clustering of samples, and 16112 genes selected after removing marginally expressed genes. Z-scores were calculates using batch effect corrected, variance stabilizing transformed expression levels of the selected genes. Marginally expressed genes created a large gap in the center of the heat map, therefore excluded from the figure. The color intensities correspond to the gene expression standard deviation from the mean. Red represents upregulation, and blue represents downregulation.


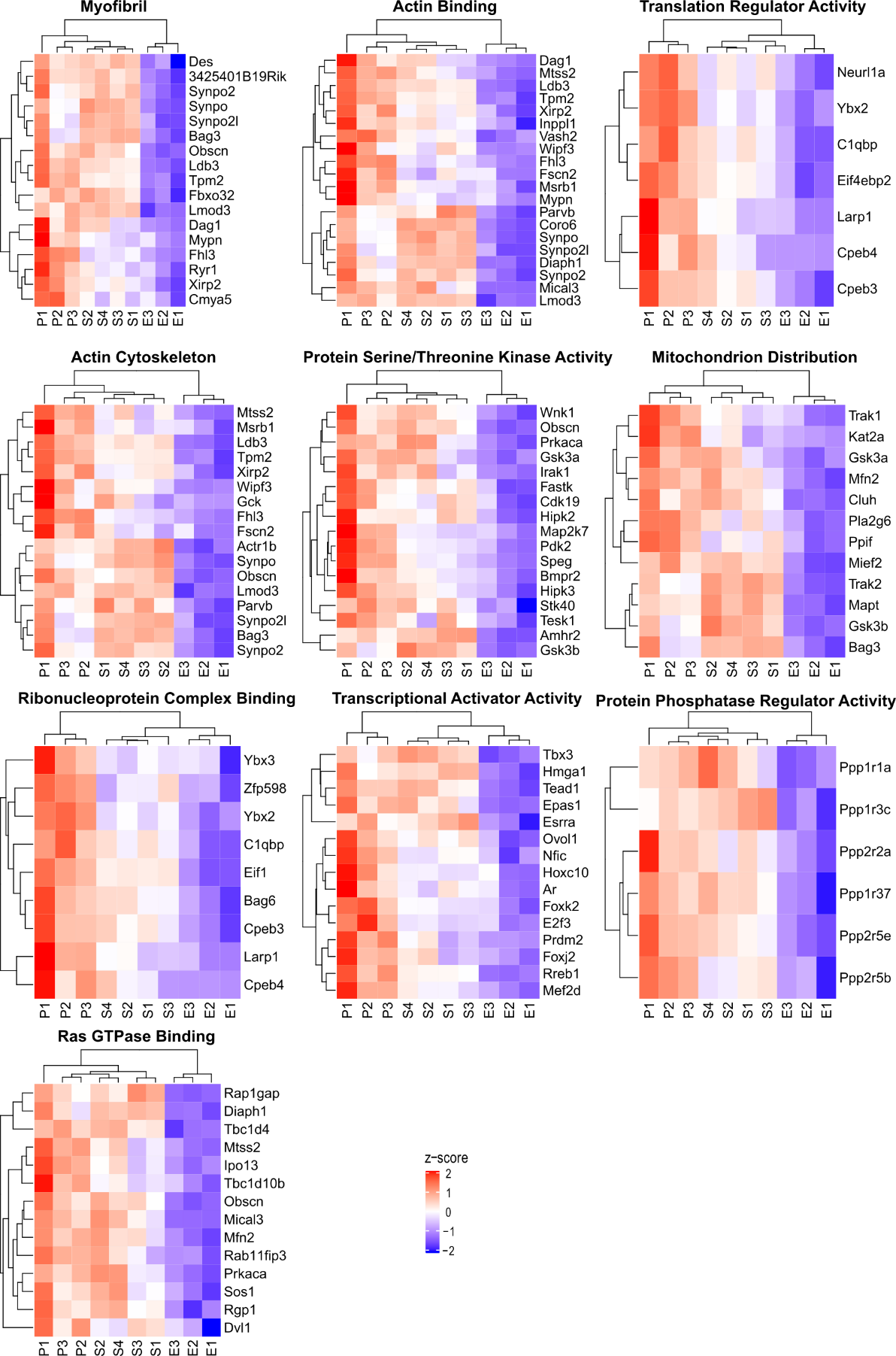


**Figure S3:** Gene expression heatmaps associated with the top 10 significant gene ontology terms enriched from the gene cluster 1, which was identified through k-means clustering of differentially expressed genes.

**Figure S4**: Gene expression heatmaps associated with the top 10 significant gene ontology terms enriched from the gene cluster 2, which was identified through k-means clustering of differentially expressed genes.
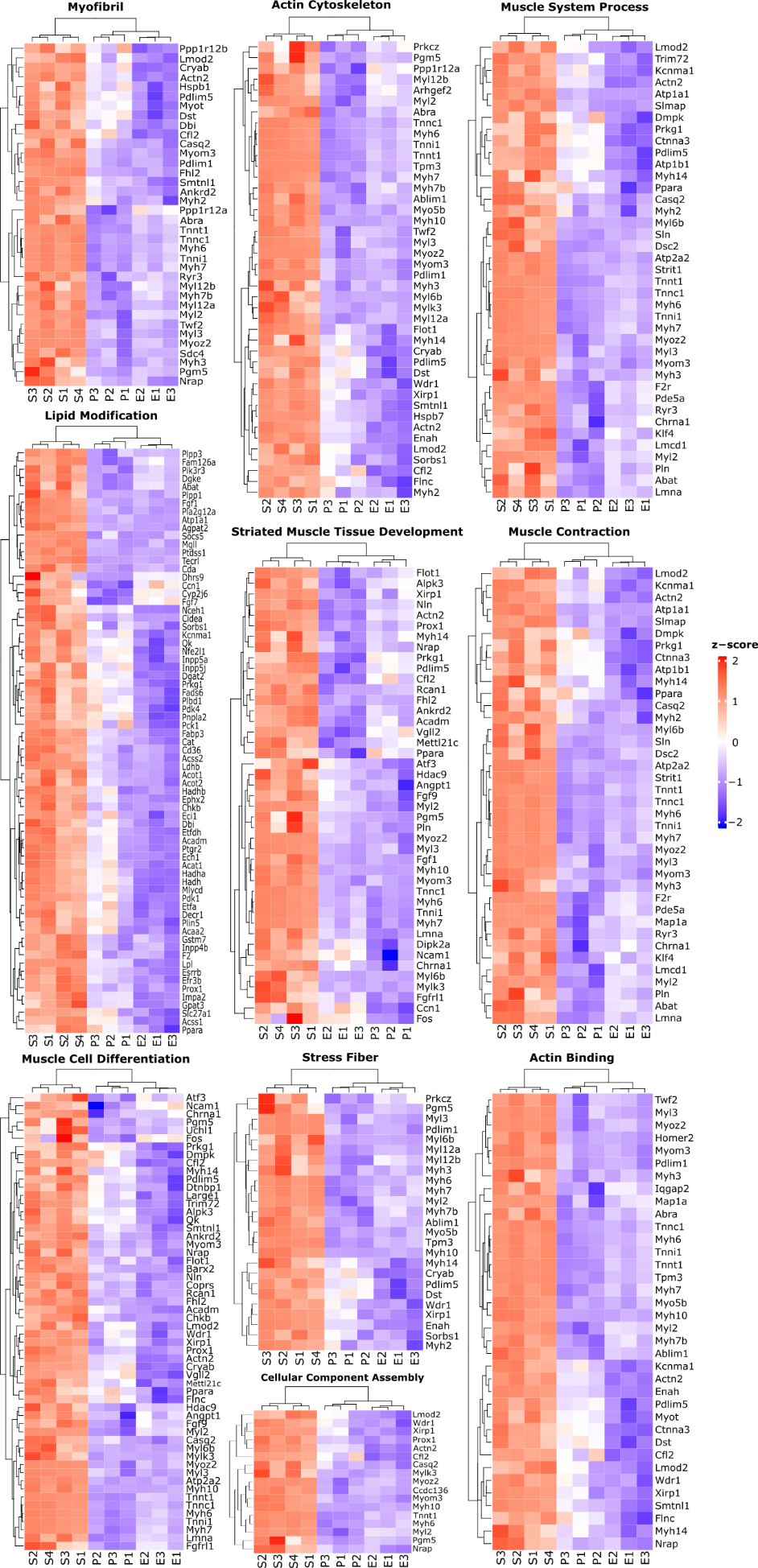


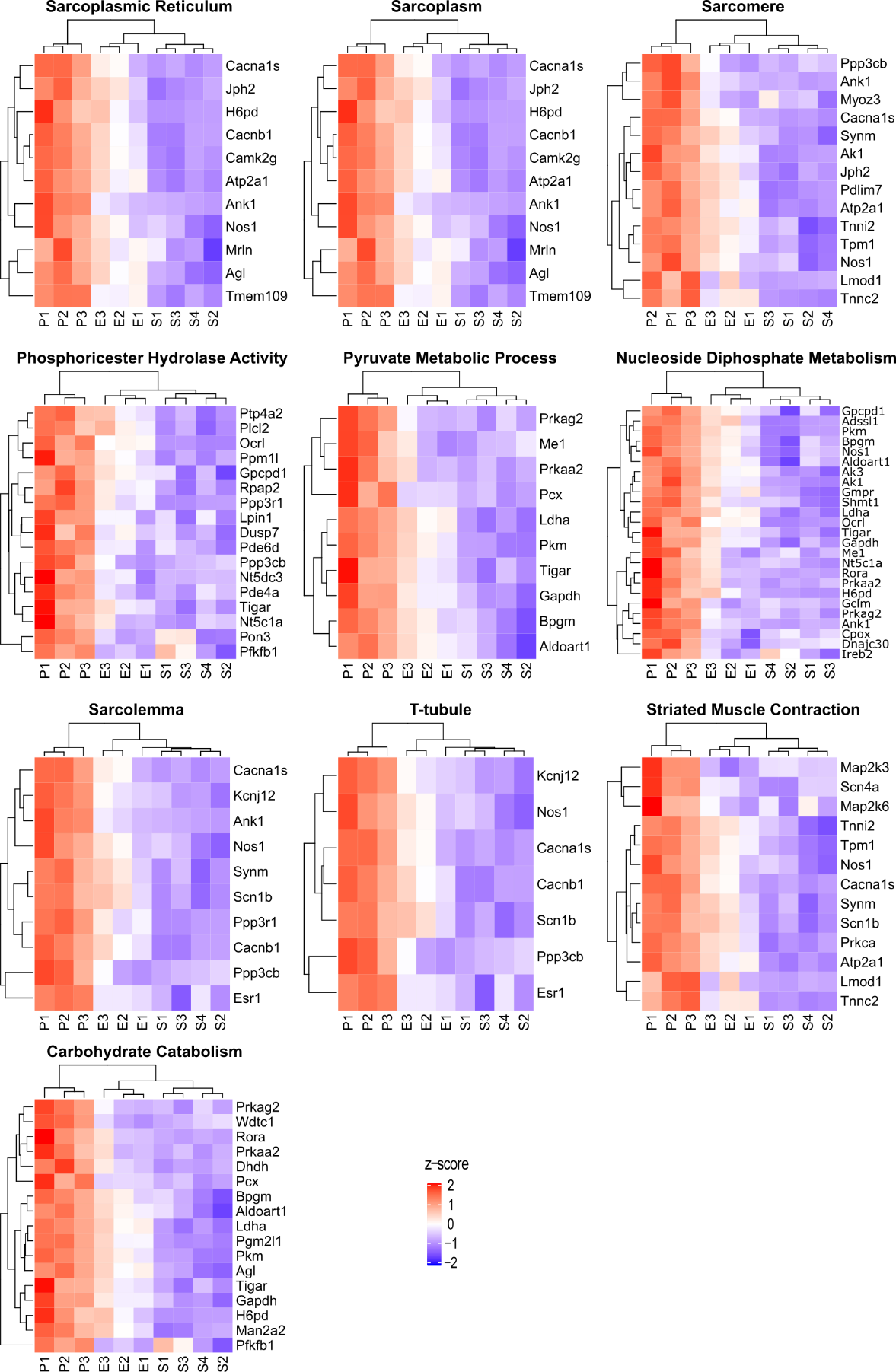


**Figure S5**: Gene expression heatmaps associated with the top 10 significant gene ontology terms enriched from the gene cluster 3, which was identified through k-means clustering of differentially expressed genes.


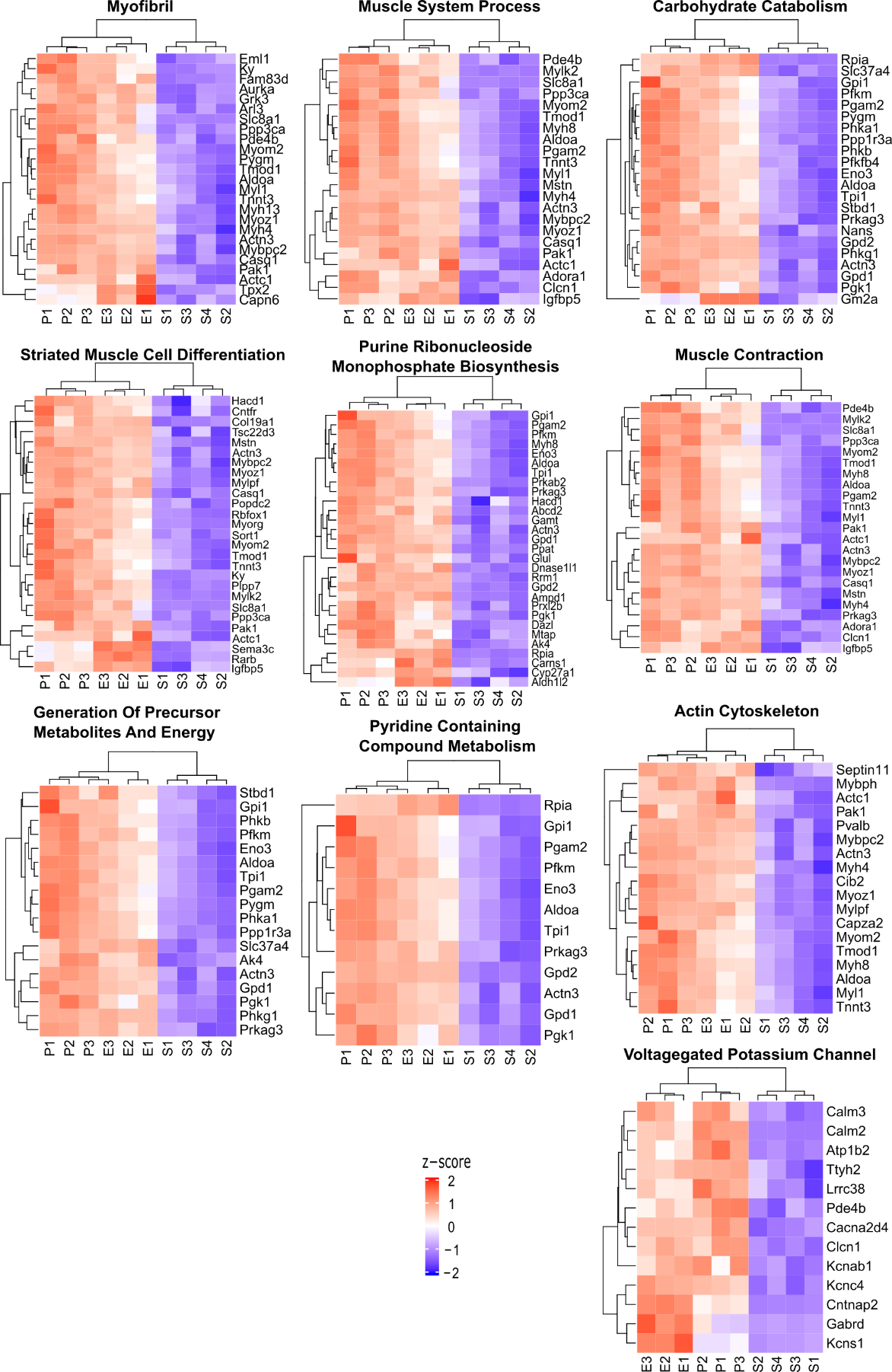


**Figure S6**: Gene expression heatmaps associated with the top 10 significant gene ontology terms enriched from the gene cluster 4, which was identified through k-means clustering of differentially expressed genes.


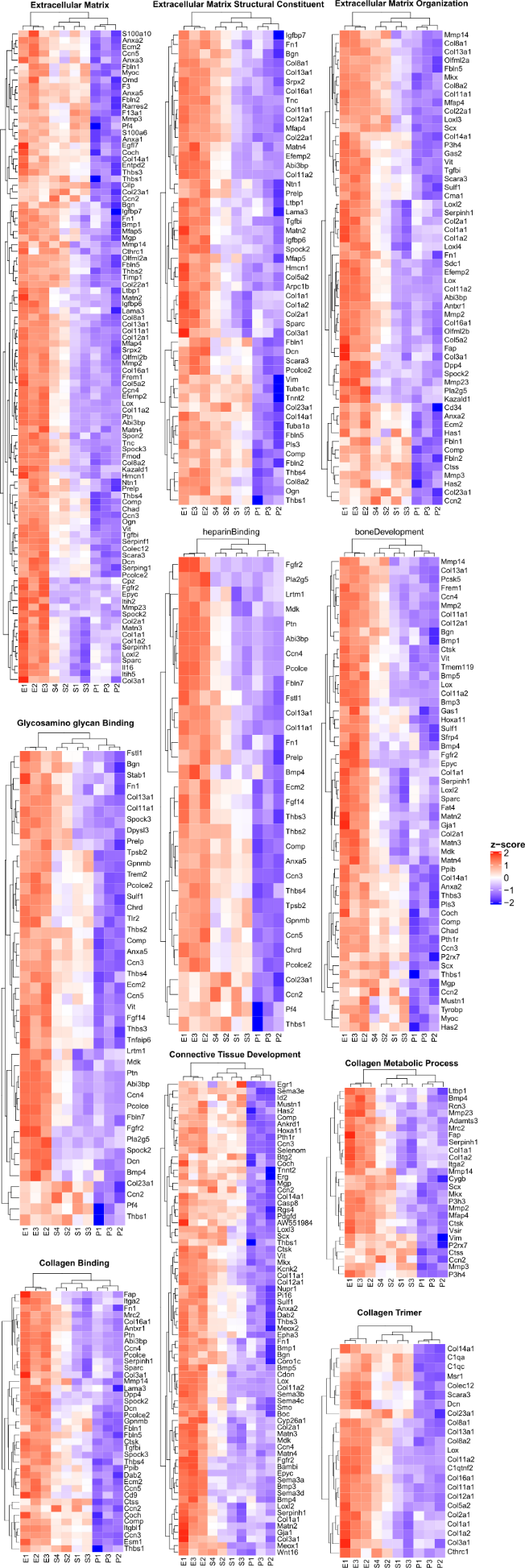
**Figure S7**: Gene expression heatmaps associated with the top 10 significant gene ontology terms enriched from the gene cluster 5, which was identified through k-means clustering of differentially expressed genes.
