# Supplementary figures and images for: Comparative analysis of the transcriptomes of EDL, psoas, and soleus muscles from mice
Source: BMC Genomics. 2020 Nov 19;21:808. doi: 10.1186/s12864-020-07225-2 (PMC7678079; doi:10.1186/s12864-020-07225-2)

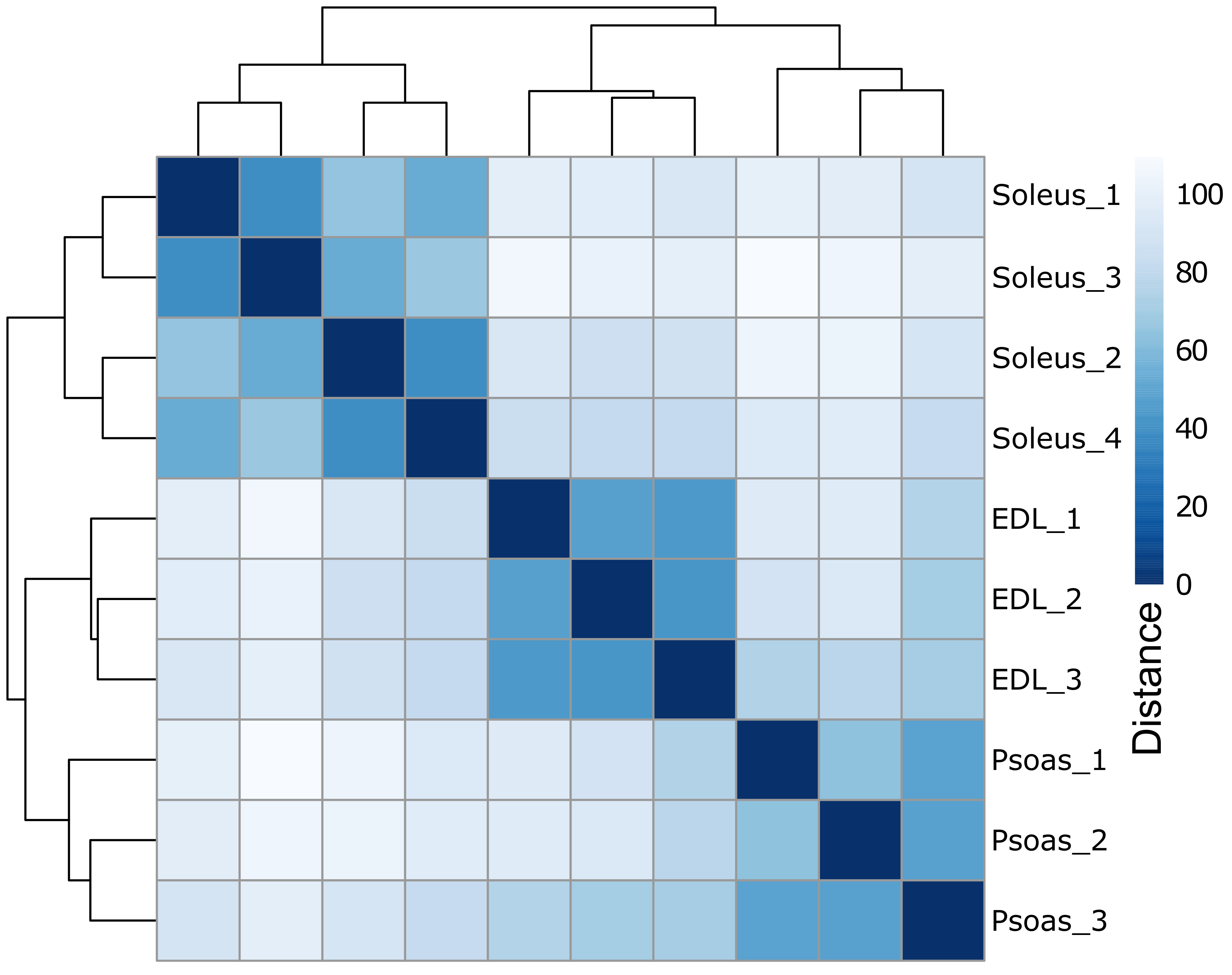

Supplement: Supplementary file 1 — Additional file 1. Additional figures and tables supporting the main text of the manuscript. Referred as SI in the main text. [file 12864_2020_7225_MOESM1_ESM.zip › Figure S1.tif]

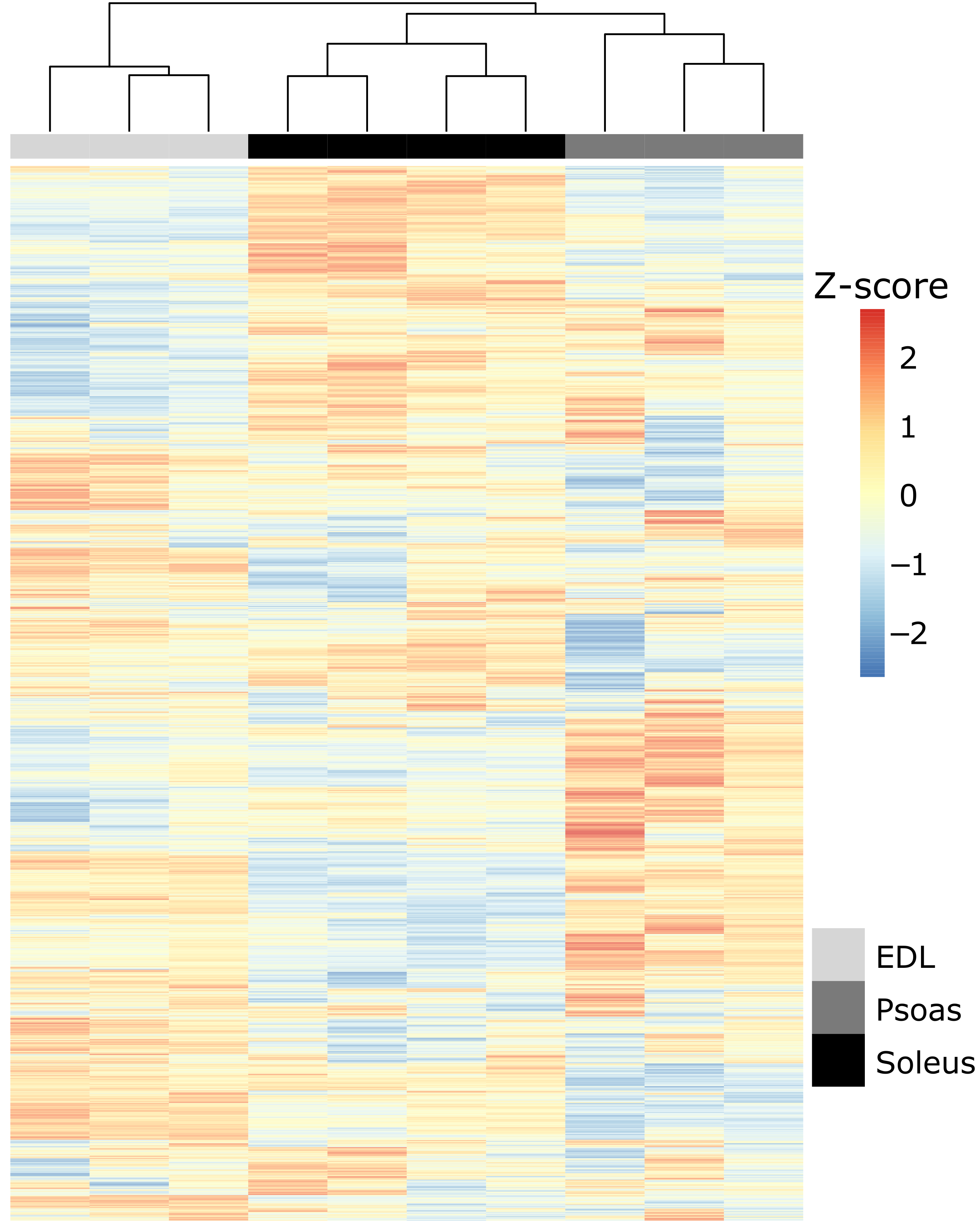

Supplement: Supplementary file 1 — Additional file 1. Additional figures and tables supporting the main text of the manuscript. Referred as SI in the main text. [file 12864_2020_7225_MOESM1_ESM.zip › Figure S2.tif]

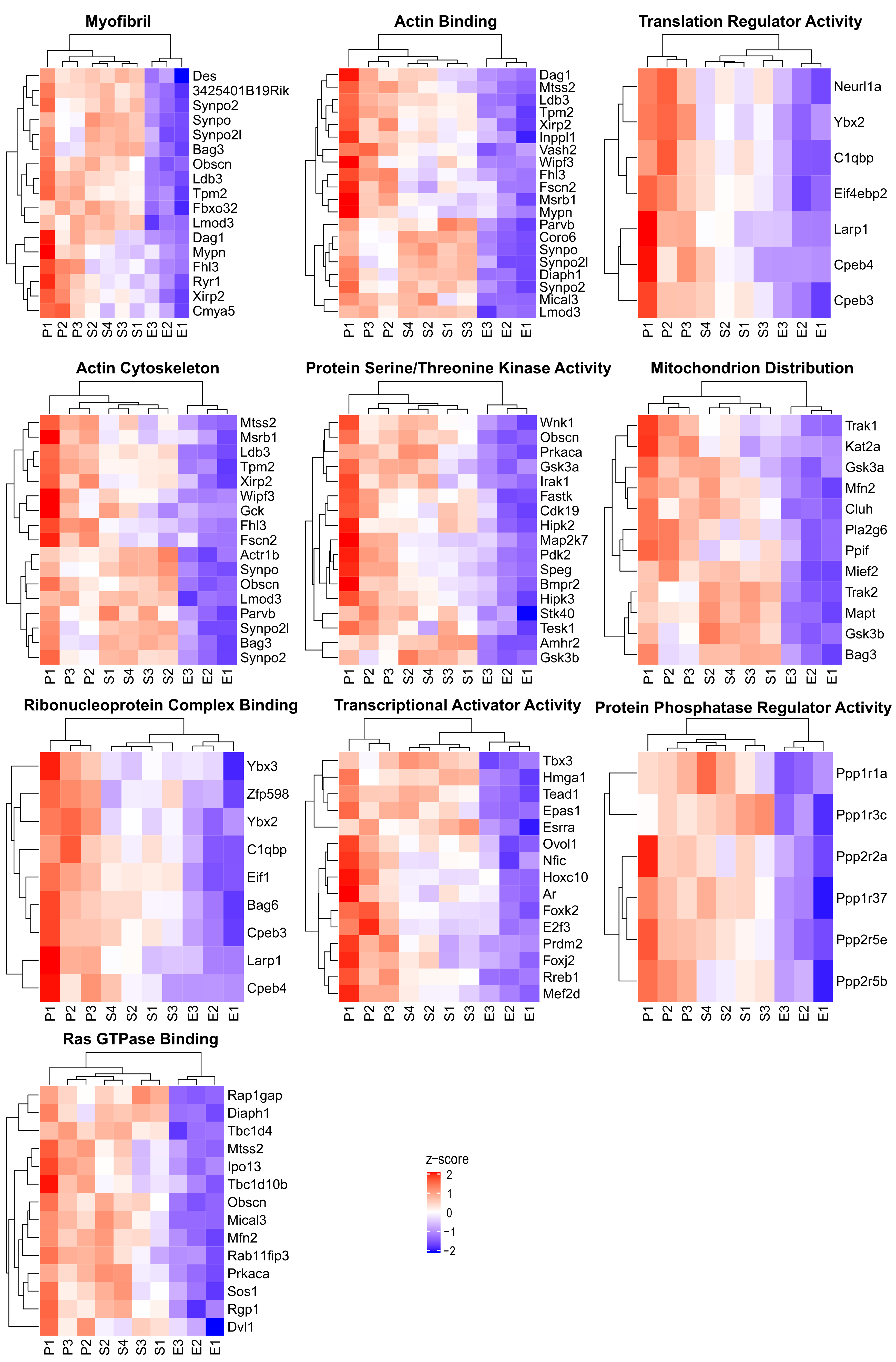

Supplement: Supplementary file 1 — Additional file 1. Additional figures and tables supporting the main text of the manuscript. Referred as SI in the main text. [file 12864_2020_7225_MOESM1_ESM.zip › Figure S3.tif]

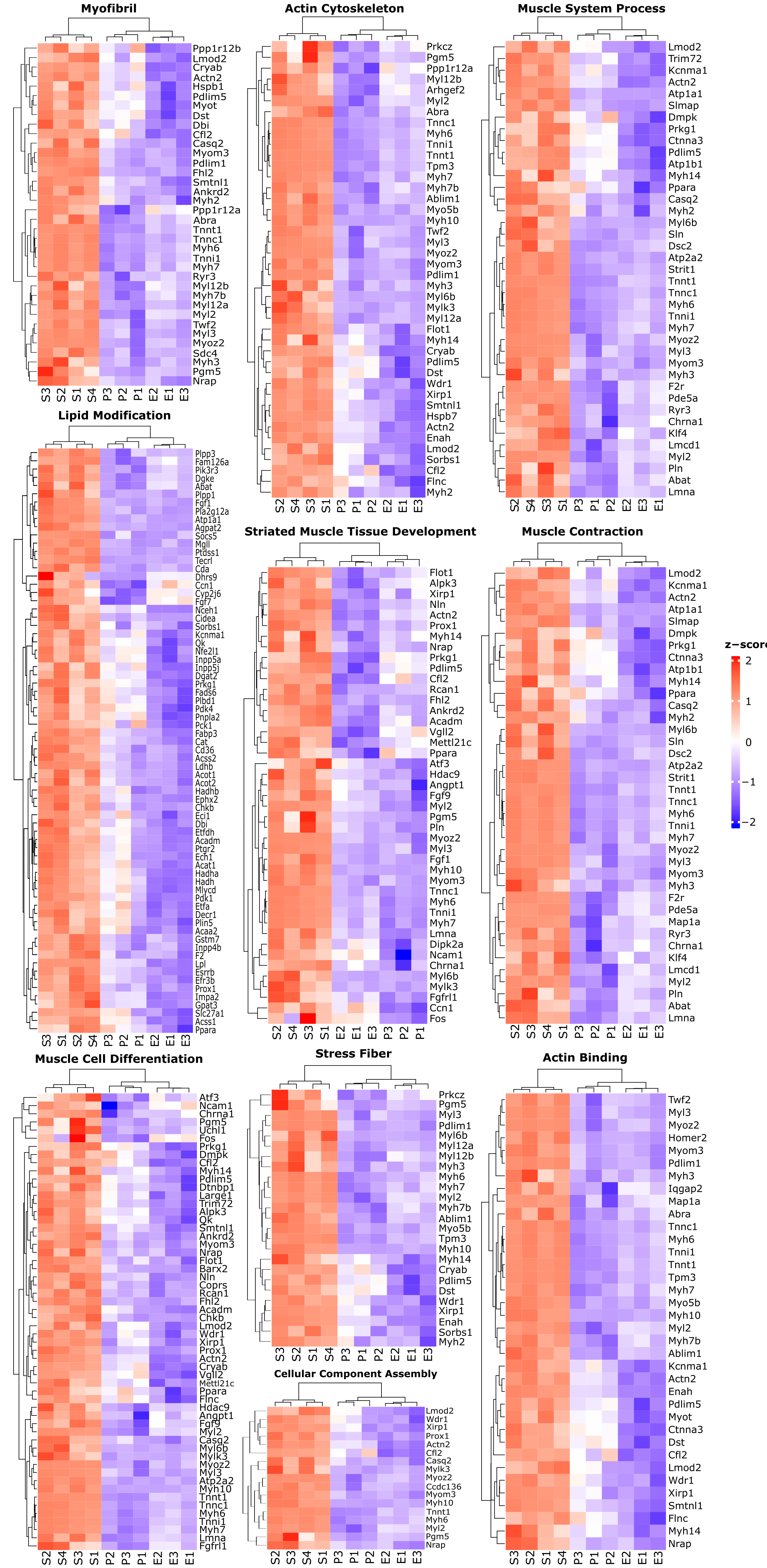

Supplement: Supplementary file 1 — Additional file 1. Additional figures and tables supporting the main text of the manuscript. Referred as SI in the main text. [file 12864_2020_7225_MOESM1_ESM.zip › Figure S4.tif]

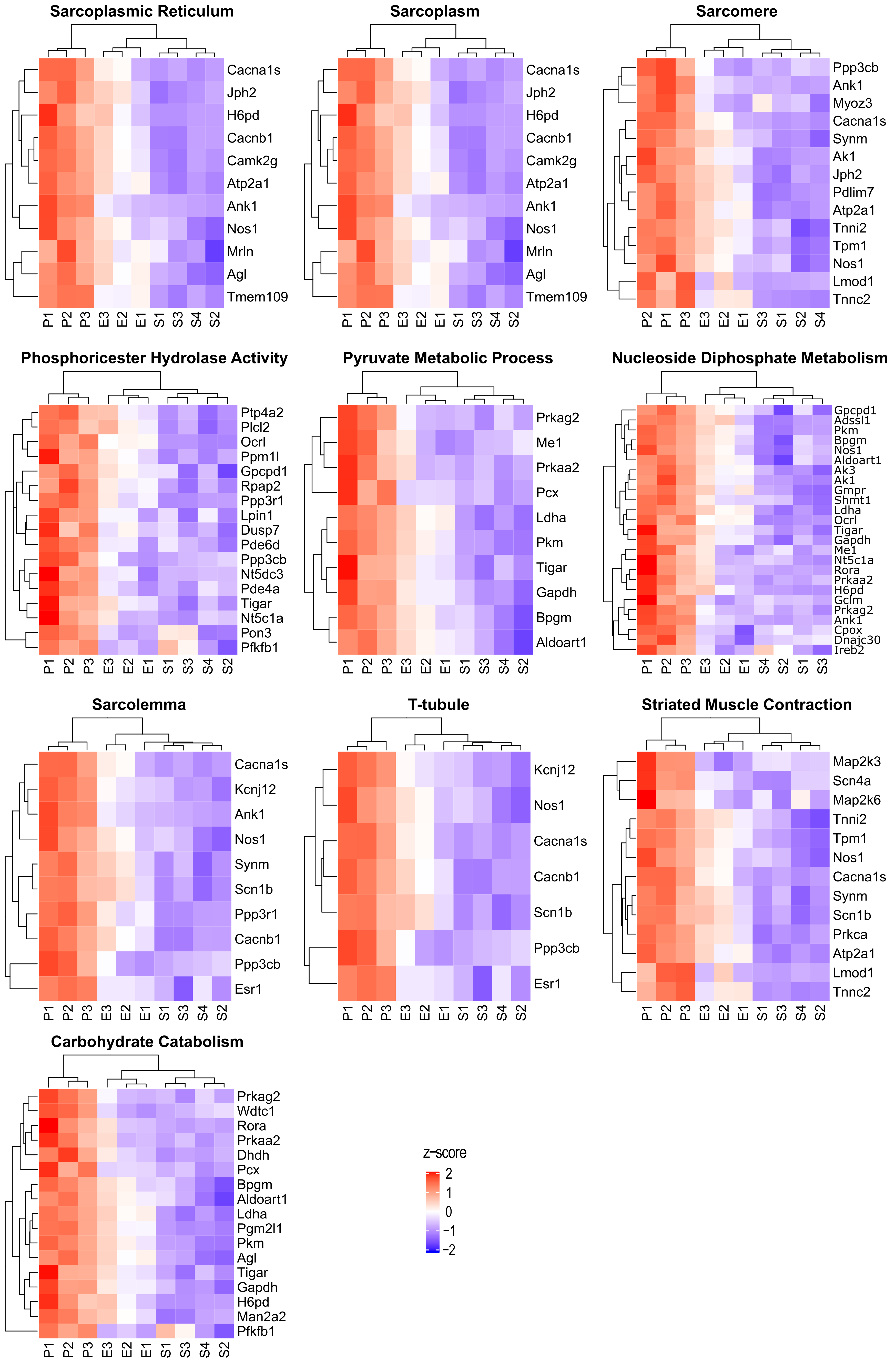

Supplement: Supplementary file 1 — Additional file 1. Additional figures and tables supporting the main text of the manuscript. Referred as SI in the main text. [file 12864_2020_7225_MOESM1_ESM.zip › Figure S5.tif]

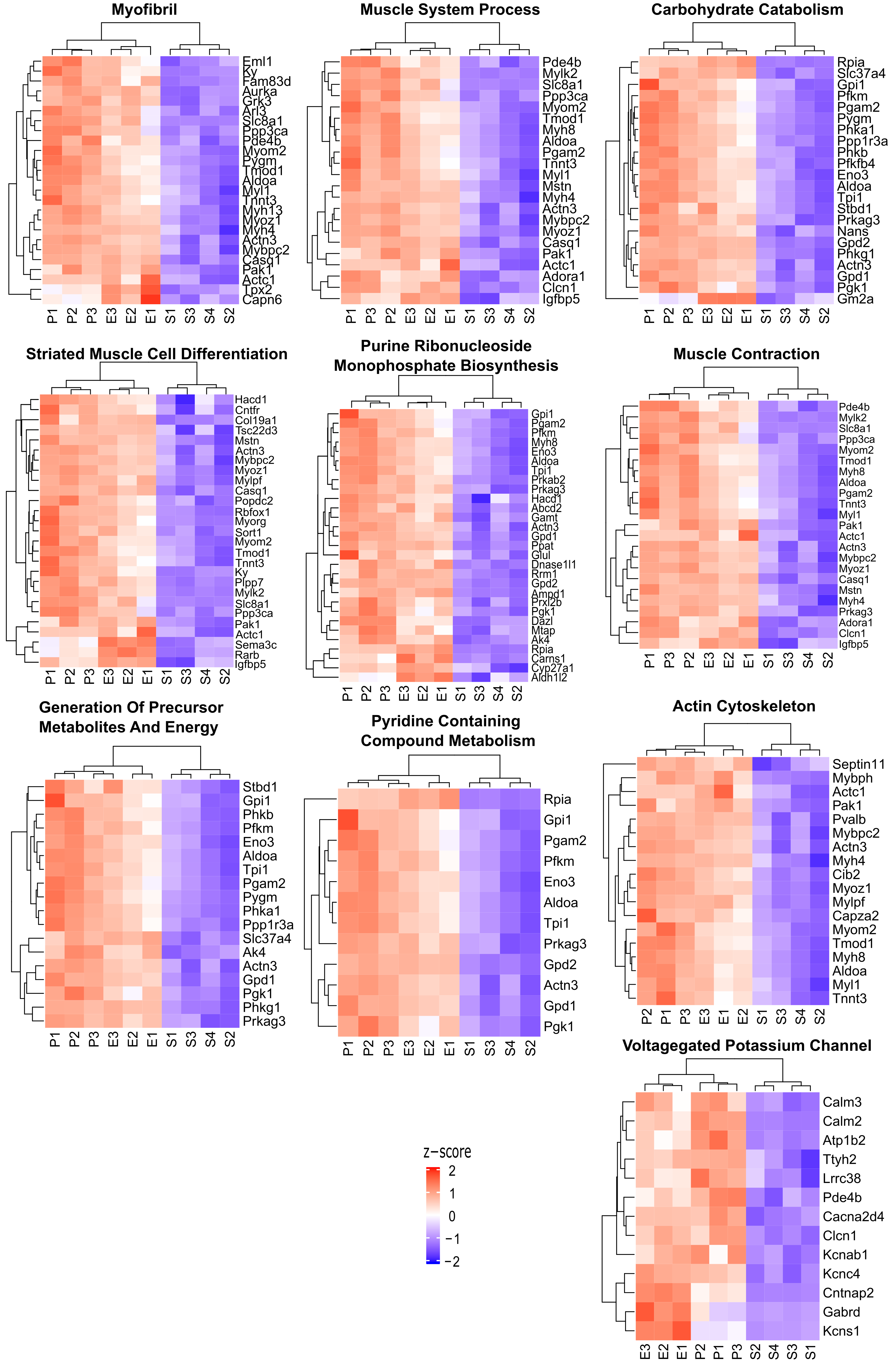

Supplement: Supplementary file 1 — Additional file 1. Additional figures and tables supporting the main text of the manuscript. Referred as SI in the main text. [file 12864_2020_7225_MOESM1_ESM.zip › Figure S6.tif]

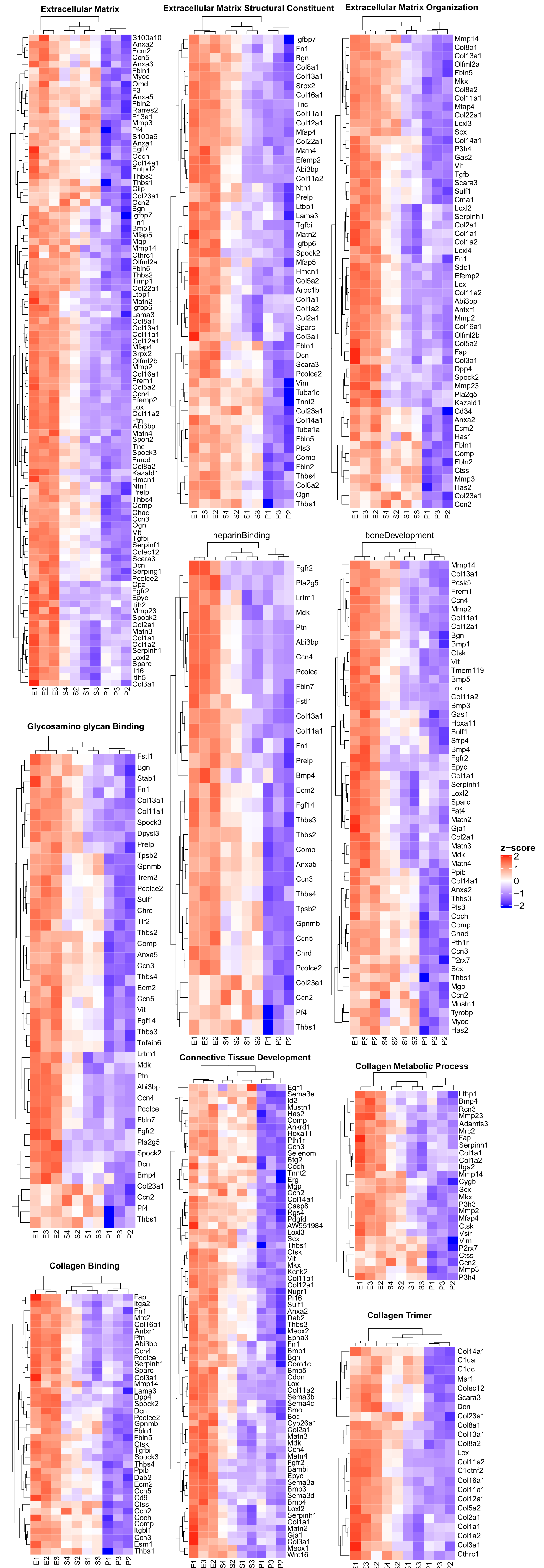

Supplement: Supplementary file 1 — Additional file 1. Additional figures and tables supporting the main text of the manuscript. Referred as SI in the main text. [file 12864_2020_7225_MOESM1_ESM.zip › Figure S7.tif]
